# Not just Rubisco: integrated strategies to overcome the biochemical limitations of C_3_ photosynthesis

**DOI:** 10.3389/fpls.2026.1854086

**Published:** 2026-07-09

**Authors:** Shivasharanappa S. Patil, Bagyalakshmi Muthan, Sanju A. Sanjaya

**Affiliations:** 1 Agricultural and Environmental Research Station and Energy and Environmental Science Institute, West Virginia State University, Institute, WV, United States; 2 Department of Biology, Agricultural and Environmental Research Station and Energy and Environmental Science Institute, West Virginia State University, Institute, WV, United States

**Keywords:** carbon partitioning, carbon-concentrating mechanisms, crop improvement, genetic engineering, triose-phosphate utilization

## Abstract

Photosynthesis is fundamental to global food security, but carbon assimilation efficiency in most C_3_ crops remains significantly below its theoretical maximum. Three principal biochemical processes limit photosynthetic performance: ribulose-1,5-bisphosphate carboxylase/oxygenase (Rubisco) catalytic activity, ribulose-1,5-bisphosphate (RuBP) regeneration via the Calvin-Benson-Bassham (CBB) cycle, and triose-phosphate utilization (TPU) for sucrose and starch biosynthesis. These constraints, which vary across environmental conditions, are closely linked through carbon metabolism, energy supply, and source–sink regulation. Recent advances in plant physiology, molecular genetics, and synthetic biology have identified new opportunities to address these limitations individually and in concert. In this review, we synthesize recent progress in understanding these three biochemical constraints and evaluate strategies to address these constraints through coordinated genetic and metabolic engineering. Future improvements in crop productivity will require integrated approaches that simultaneously optimize carboxylation capacity, electron transport, and end-product utilization while maintaining metabolic equilibrium in crops under field conditions.

## 1 Introduction

In light of the rapidly changing environment and increasing global food demands, improving photosynthetic efficiency has become a pressing scientific and humanitarian priority (Long et al., 2016). Photosynthesis is the biochemical process by which plants convert water and CO_2_ into carbohydrates and oxygen using light as energy. This process occurs via two stages: the photochemical and biochemical reactions. During the photochemical reaction, the photolysis of water produces oxygen, protons, and electrons. This process generates ATP and NADPH, which are utilized in the biochemical reactions to fix CO_2_ into carbohydrates. The efficiency of converting this light energy into chemical energy varies across the three major photosynthetic pathways, C_3_, C_4_, and CAM (Crassulacean acid metabolism), each requiring distinct, specialized leaf ultrastructures (Hartzell et al., 2018).

Photosynthesis in C_3_ plants follows the core Calvin-Benson-Bassham (CBB) cycle in which atmospheric CO_2_ diffuses through stomata into mesophyll cells and is fixed into 3-phosphoglycerate by the world’s most abundant enzyme: ribulose-1,5-bisphosphate carboxylase/oxygenase (“Rubisco”). Approximately 90% of agricultural crops depend on the C_3_ pathway, but its operational efficiency under field conditions is typically half of the theoretical maximum (Zhu et al., 2008; 2010). This inefficiency arises from the dual carboxylase and oxygenase activities of Rubisco. Relative increases in the oxygenase activity of Rubisco, particularly at high temperature, promote photorespiratory carbon loss, a process in which Rubisco fixes O_2_, producing glycolate and releasing CO_2_ through the photorespiration pathway (Brown et al., 2011). By contrast, C_4_ and CAM plants employ specialized mechanisms for concentrating CO_2_ involving spatial (in C_4_ plants) and temporal separation (in CAM plants). In both systems, phosphoenolpyruvate carboxylase (PEPC) initiates CO_2_ fixation, increasing the CO_2_ concentration around Rubisco and thereby decreasing photorespiration (Moreno-Villena et al., 2022). There is a growing trend to combine the inherent advantages of the C_4_ and CAM mechanisms to enhance photosynthetic efficiency and productivity in C_3_ crops (Yang et al., 2024).

Although crop yields have traditionally been increased by improving the harvest index (the ratio of harvestable yield to biomass) and agronomic practices (Cui et al., 2021), accumulating evidence indicates that targeted genetic and physiochemical interventions could overcome key bottlenecks and significantly enhance photosynthetic efficiency. Deploying such strategies requires a comprehensive understanding of the biochemical reactions at the core of photosynthesis in C_3_ crops and how they are regulated.

In C_3_ plants, photosynthetic efficiency is constrained by three major biochemical limitations: 1) carboxylation, or Rubisco-mediated carbon assimilation; 2) ribulose-1,5-bisphosphate (RuBP) regeneration, or electron transport; and 3) triose-phosphate utilization (TPU) based on the inorganic phosphate (Pi) supply (Sharkey et al., 2007; von Caemmerer, 2013). These limitations, which operate across varying internal CO_2_ concentrations and environmental conditions, are tightly interconnected, regulating overall carbon flux through the photosynthetic apparatus. In general, the Rubisco-based limitation of photosynthesis occurs at low intercellular CO_2_ (C*_i_*) concentrations due to the low catalytic turnover rate of Rubisco and poor specificity for CO_2_ over O_2_ (Sharkey et al., 2007). At intermediate C*_i_* levels, the limitation shifts to the regeneration of RuBP, which depends on electron transport driven by photochemical reactions and is modulated by ATP/NADPH ratios and external stress factors (McClain et al., 2023). Under elevated CO_2_ or low O_2_ conditions, limited triose-phosphate use may dominate due to insufficient sink capacity to process the fixed carbon into end products such as sucrose and starch (McClain et al., 2023).

**Figure 1:**
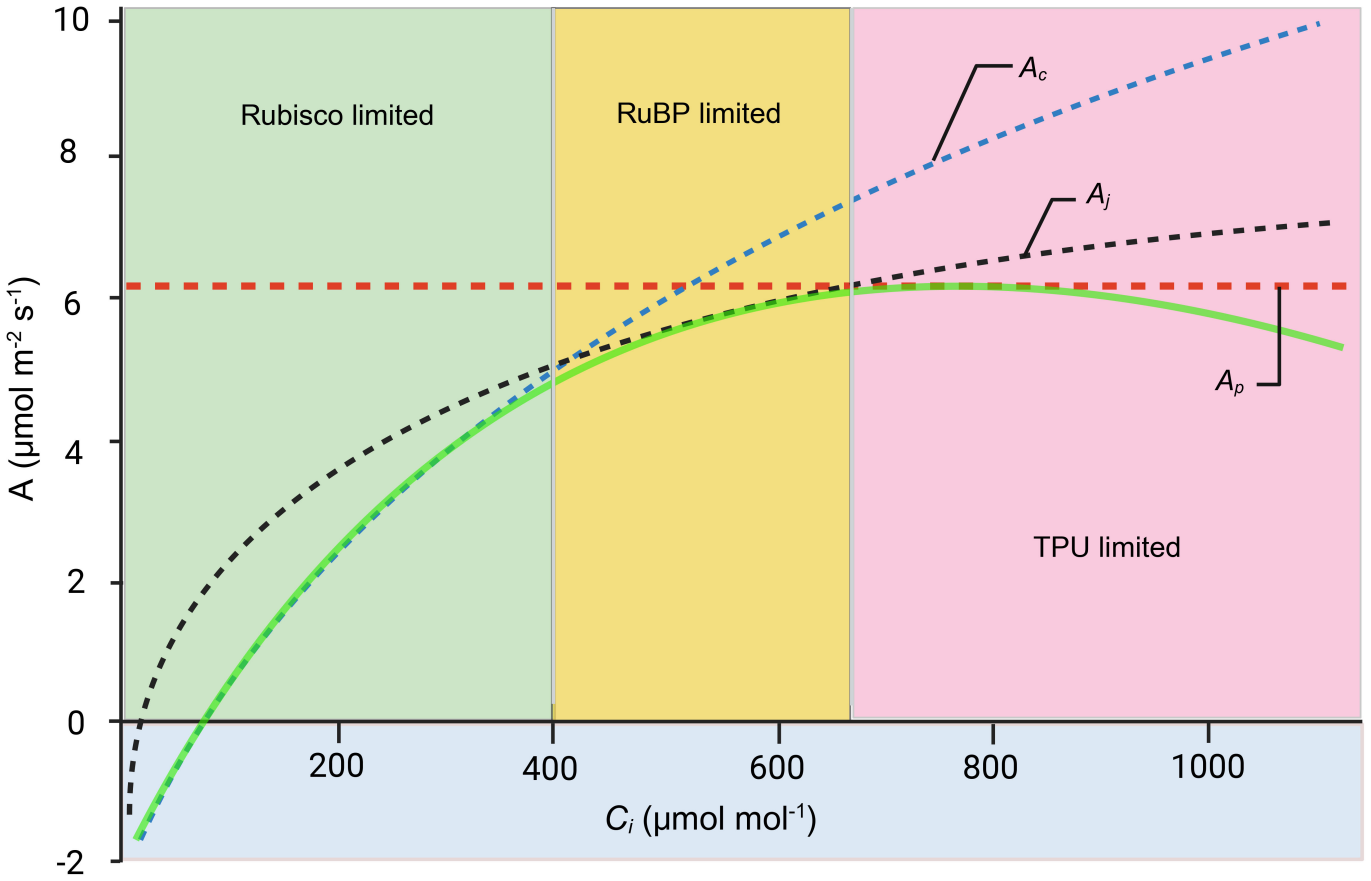
Representative *A/C_i_*, curve showing three biochemical limitations of C_3_ photosynthesis. The blue dashed line represents Rubisco-limited assimilation (*A_c_*) operating at lower intercellular CO_2_ concentration (*C_i_*). At intermediate *C_i_*, RuBP regeneration limited assimilation (Aj) dominates, as represented by black dot lines. The horizontal red dashed line represents triose phosphate utilization limitation (*Ap*) operating at high *C_i_*. The green curve (solid) shows the observed net assimilation rate. The vertical lines differentiate the limitation transitions observed with increasing *C_i_*, and the light blue area represents respiration at lower CO_2_ concentration. This figure created in BioRender uxhfcim.

The limitations on photosynthesis can be quantified using A/C*_i_* response curves, in which the net CO_2_ assimilation rate (A) is plotted against C*_i_* concentration. Such curves are generated using infra-red gas analyzers (Long and Bernacchi, 2003; Sharkey et al., 2007). The Farquhar-von Caemmerer-Berry (FvCB) model uses data from these curves to mechanistically integrate the chemical kinetics of Rubisco carboxylation and oxygenation reactions, electron transport associated with the CBB cycle, and TPU (Farquhar et al., 1980) to identify the potentially limiting parameters: Vc*_max_* (maximum carboxylation capacity of Rubisco), J*_max_* (maximum electron transport rate), and TPU capacity (von Caemmerer, 2013). Complementary diagnostic tools, such as measuring chlorophyll fluorescence, can clarify which of these phases is limiting (Sharkey et al., 2007; Fabre et al., 2019). Three limitation states are also reflected in the A/C*_i_* curve (**Figure 1**), providing valuable physiological insights into the factors governing photosynthetic performance in crops.

The capacity for RuBP regeneration can be enhanced by overexpressing genes encoding CBB enzymes (Lefebvre et al., 2005; Driever et al., 2017), improving components of the photosynthetic electron transport chain (Yamori et al., 2011; Ermakova et al., 2024), or modulating ATP synthase activity (Yamori and Shikanai, 2016) to increase the supply of ATP. To address constraints on TPU, carbon sink strength can be increased by improving phloem loading, enhancing sucrose export, and engineering more efficient phosphate recycling (McClain and Sharkey, 2023). Beyond these core biochemical programs, diffusional conductance from stomata (g_m_) and mesophyll cells (g_m_) also regulates internal CO_2_ availability and thus influences the overall effectiveness of any strategy aimed at enhancing photosynthesis (Farquhar and Sharkey, 1982; Adachi et al., 2013; Gago et al., 2016).

Although numerous reviews have addressed possible biotechnological approaches to improve photosynthesis, we reasoned that organizing these strategies according to the specific limiting step they target would provide a much-needed mechanistic framework. This perspective supports the logical application of synthetic biology and advanced molecular tools, thereby enabling the coordinated integration of multiple interventions to enhance overall photosynthetic capacity. In this review, we classify and evaluate strategies for improving photosynthetic efficiency in C_3_ plants based on the key biochemical limitations they address. We highlight recent biotechnological advances, discuss associated trade-offs and challenges, and propose integrated approaches for translating these strategies into field-level improvements.

## 2 Biochemical limitation: carboxylation efficiency

Rubisco (ribulose-1,5-bisphosphate carboxylase/oxygenase), the most abundant protein in plants, catalyzes the carboxylation of RuBP with CO_2_ in the CBB cycle, producing two molecules of 3-phosphoglycerate (Farquhar et al., 1980). As the primary gateway for the assimilation of inorganic carbon into organic compounds, Rubisco plays a central role in determining net photosynthetic carbon gain in C_3_ plants (von Caemmerer and Quick, 2000). However, the relatively slow catalytic rate and competing oxygenase activity of Rubisco impose a major biochemical constraint on photosynthetic efficiency. Consequently, enhancing carboxylation efficiency has become a major target for biotechnological interventions aimed at improving crop productivity. Approaches to overcome this limitation include engineering Rubisco variants with improved catalytic turnover rates and/or enhanced specificity for CO_2_ (Whitney et al., 2015; Martin-Avila et al., 2020), manipulating other enzymes of the CBB cycle, and introducing carbon-concentrating mechanisms (CCMs) such as cyanobacterial carboxysomes that elevate CO_2_ concentrations in the vicinity of Rubisco and reduce photorespiratory losses (Whitney et al., 2015; Martin-Avila et al., 2020). Similar strategies include engineering algal pyrenoids, implementing photorespiratory bypass pathways, and enhancing the activity of Rubisco activase (RCA) and other Rubisco-associated proteins that maintain Rubisco functionality under environmental stresses such as elevated temperatures (>35 °C), high CO_2_, or low O_2_ conditions (Crafts-Brandner and Salvucci, 2000; Yamori et al., 2012). Collectively, these approaches seek to increase the efficiency of carbon fixation and thereby improve photosynthetic performance and crop yield.

Efforts to improve Rubisco kinetics frequently encounter trade-offs: a higher catalytic rate is often accompanied by lower specificity for CO_2_, which can increase oxygenation and photorespiration under ambient CO_2_ conditions (Tcherkez et al., 2006; Iniguez et al., 2021). Indeed, enhancing catalytic speed by lowering activation barriers for carboxylation typically also lowers the barriers to oxygenation, leading to metabolic penalties such as increased photorespiratory flux, higher energy costs, and potentially slower plant growth (Bar-Even, 2018). These limitations are especially pronounced under current atmospheric CO_2_ concentrations (420 ppm) and abiotic stress conditions such as heat, drought, or fluctuating light, where the carboxylation capacity of Rubisco, commonly expressed as Vc*_max_*, often constrains photosynthesis in C_3_ crops (Mueller-Cajar and Whitney, 2008; Whitney et al., 2015). Enhancing Rubisco biogenesis remains a significant challenge because it requires the tightly coordinated expression of nucleus-encoded and plastid-encoded genes, along with a suite of molecular chaperones and assembly factors. Moreover, Rubisco represents a considerable nitrogen investment, accounting for up to 30% of total leaf nitrogen in many C_3_ species (Makino and Osmond, 1991; Parry et al., 2013; Evans and Clarke, 2019).

Carboxylation capacity (Vc*_max_*) is a core parameter of the FvCB model of C_3_ photosynthesis (Farquhar et al., 1980), which estimates the upper limit of CO_2_ assimilation based on the abundance and kinetic properties of Rubisco. Higher Vc*_max_* values are associated with a 5–15% gain in C_3_ photosynthetic performance and biomass accumulation, particularly under elevated CO_2_ or heat-stress conditions (Zhu et al., 2010; Walker et al., 2016). A comprehensive dataset compiling *in vitro* Rubisco kinetic measurements from nearly 300 organisms with Form I Rubisco (Predominant Rubisco isoform) reveals limited natural variation in Rubisco kinetics. However, the carboxylation rate (k_cat,C_) of >95% of organisms ranges from 1–10 s^−1^ (Bouvier et al., 2024; de Pins et al., 2025), and the preference for CO_2_ over O_2_ (S_C/O_) varies by <10% among C_3_ plants (Flamholz et al., 2019). Although the variation is limited, it is not evolutionarily fixed. Rubisco continues to undergo gradual refinement toward improved catalysis and CO_2_ assimilation, but at an exceptionally slow pace compared with most other enzymes (Bouvier et al., 2024). This limitation coupled with inherent catalytic trade-offs has constrained progress through conventional breeding approaches (Tcherkez et al., 2006), motivating the development of biotechnological strategies.

### 2.1 Engineering Rubisco to enhance Vc_max_: assembly, activation, and optimized synthesis

Strategies aimed at enhancing Vc*_max_* include overexpressing Rubisco small subunit genes (*RbcS*) to promote holoenzyme assembly (Suzuki et al., 2007), introducing Rubisco isoforms from cyanobacteria or algae combined with carbon-concentrating mechanisms (CCMs) such as the use of carboxysomes and bicarbonate transporters to suppress photorespiration (Lin et al., 2014; Hanson et al., 2016), and improving Rubisco activation using thermostable variants of RCA (Kurek et al., 2007). The successful heterologous expression of Rubisco subunit genes in land plants also requires the coordinated co-expression of genes encoding key chaperones and assembly factors, including *RUBISCO ACCUMULATION FACTOR 1* (*RAF1*), *RUBISCO LARGE−SUBUNIT ASSEMBLY CHAPERONE* (*RbcX*), *BUNDLE−SHEATH DEFECTIVE 2* (*BSD2*), and the Chaperonin 60 (Cpn60)–Cpn20 complex, to ensure proper folding and catalytic function (Whitney et al., 2015; Wilson et al., 2016).

In major crops such as rice and maize (*Zea mays*), enhancing Rubisco content increases the demand for leaf nitrogen but also increases canopy carbon gain by 10–15%, thus offsetting the metabolic cost of this additional nitrogen investment (Paul, 2021). Although Rubisco accounts for a substantial fraction of leaf nitrogen, much of this nitrogen is remobilized during leaf senescence and recycled to developing grains, thereby mitigating whole-plant nitrogen costs (Evans and Clarke, 2019). Furthermore, overexpressing Rubisco assembly genes such as *RAF1* and *BSD2* improves holoenzyme stability and minimizes the accumulation of misfolded or inactive Rubisco, enhancing nitrogen-use efficiency associated with increased Rubisco content (Vitlin Gruber and Feiz, 2018).

More advanced approaches involve the directed evolution and synthetic design of Rubisco using heterologous systems such as *Escherichia coli* to generate and screen enzyme variants with improved specificity, catalytic stability, and performance under stress conditions (Wilson et al., 2018). These efforts are often complemented by additional innovations, including the introduction of photorespiratory bypass pathways, optimization of CBB enzymes (Raines, 2011; Simkin et al., 2015), modification of canopy architecture to improve light distribution (Drewry et al., 2014), and incorporation of natural or synthetic CCMs (Furbank et al., 2009; Long et al., 2016). In the next section, we explore recent advances in Rubisco-targeted engineering and processes aimed at synergistically enhancing photosynthetic capacity and crop productivity.

### 2.2 Rubisco engineering and replacement

Increasing the abundance of Rubisco has proven effective in enhancing photosynthetic capacity when the nitrogen supply is adequate (Salesse-Smith et al., 2025). Rubisco functions as a hexadecameric holoenzyme (L_88_) comprising eight large subunits (RbcL) encoded by the plastid genome and eight small subunits (RbcS) encoded by a nuclear multi-gene family. RbcS is rate limiting in most C_3_ crops under ambient CO_2_ conditions, while excess RbcL that fails to assemble with sufficient RbcS may accumulate and degrade rather than forming functional Rubisco (Liu et al., 2010; Vitlin Gruber and Feiz, 2018). Most engineering strategies prioritize smaller subunits with some assembly chaperones, while the translation of larger subunits naturally adjusts via plastid feedback, avoiding stoichiometric imbalance (Mao et al., 2022).

Elevated Rubisco levels can be achieved through heterologous expression or CRISPR-based promoter editing and the removal of upstream open reading frames, enabling transgene-free upregulation of Rubisco subunit gene expression in crops (Si et al., 2020; Tang and Zhang, 2023). For example, transgenic rice lines with higher *RBCS* expression accumulated 25–30% more Rubisco than non-transgenic control plants and exhibited yield gains under adequate nitrogen supply; notably, yield gains were less pronounced under nitrogen-limited conditions (Yoon et al., 2020). Co-expressing *RBCS* and *RBCL* in maize had no discernable impact on Rubisco content. However, the addition of the Rubisco assembly chaperone gene *ZmRAF1* led to a >30% increase in Rubisco content, resulting in a 15% increase in the CO_2_ assimilation rate (Salesse-Smith et al., 2018, 2020, 2025).

Simply increasing Rubisco content does not always translate into improved carbon assimilation, as Rubisco activity is highly sensitive to elevated temperatures (40–45 °C), suboptimal nitrogen availability, and other abiotic stress factors (Salvucci and Crafts-Brandner, 2004). Moreover, the overproduction of Rubisco is often accompanied by a decline in Rubisco activation. An extensive review has been conducted on the activation state of Rubisco, defined as the proportion of total Rubisco in a leaf that is catalytically active and capable of RuBP carboxylation and oxygenation (Amaral et al., 2024). ATP hydrolysis by RCA drives the conformational remodeling of Rubisco, facilitating the release of inhibitory sugar-phosphate compounds (*e.g.*, RuBP, CA1P, and other tightly bound phosphorylated intermediates) from the active site to restore its carboxylation activity (Degen et al., 2021).

This becomes especially critical under heat stress and dynamic light environments, where both ATP availability and enzyme stability are compromised, in addition to the strong accumulation of inhibitory sugar phosphates. Under these circumstances, RCA activity is physiologically essential for remodeling Rubisco to reopen blocked catalytic sites. However, thermosensitivity differs among the RCA isoforms (α and β) generated through alternative splicing of its transcripts (Amaral et al., 2024). Among the α and β isoforms in wheat (*Triticum aestivum*; the Rca1b, Rca2b, and Rca2a isoforms), Rca1b is the most thermostable form but has a lower activation rate due to increased ATPase activity. Heterologous expression of the maize RCA β-isoform enhanced photosynthesis and yield in rice at high temperatures (Yamori et al., 2012; Scafaro et al., 2018). By contrast, in another study, heterologously expressing maize RCA in rice increased the activation of Rubisco but reduced total Rubisco contents, likely because activated Rubisco is less protected by sugar-phosphate inhibitors such as CA1P (2-Carboxy-D-arabitinol 1-phosphate) and RuBP, making it vulnerable to proteolytic degradation (Fukayama et al., 2018).

Plants safeguards Rubisco in an inactivated state in the dark when photosynthesis is not operating. Here, low ATP levels limit RCA activity, allowing CA1P and RuBP to bind to and inactivate Rubisco but keeping it structurally stable. This inhibitor-bound inactive state prevents unnecessary catalytic activity and protects Rubisco from proteolytic degradation by limiting its structural exposure (Sharkey, 2023). However, CA1P levels in the dark vary considerably among plant species. An attempt was made to enhance Rubisco activity by removing CA1P by overexpressing *CA1Pase* in wheat (Lobo et al., 2019). This approach unexpectedly reduced Rubisco content as well as total grain yield. Eliminating CA1P increased the vulnerability of Rubisco to proteolytic degradation due to the loss of protective inhibitor binding. This outcome is consistent with reports on RCA overexpression, where the enhanced activation state of Rubisco was also associated with reduced Rubisco abundance. Rubisco modifications must be co-engineered using strategies that enhance ATP availability through electron transport in the thylakoid, as RCA activity is also dependent on ATP hydrolysis (Scafaro et al., 2023). These findings also demonstrate that simply increasing the activation of Rubisco is counterproductive, as chaperones or protective mechanisms must be employed to stabilize Rubisco and its activation state.

Co-overexpressing thermostable maize *RCA* together with *RBCS* in rice helped maintain an adequate ATP supply from the electron transport chain and enhanced CO_2_ assimilation, increasing biomass by 26% under heat (40 °C) stress (Qu et al., 2021). Overexpressing this combination of genes (*RBCS/RCA*) in rice helped maintain higher Rubisco activation states through RCA activity. Sustained high Rubisco content through RBCS facilitated Rubisco assembly at higher temperatures (32–36 °C), resulting in improved photosynthesis (Suganami et al., 2021).

Manipulating the levels of Rubisco assembly factor levels provides another route for enhancing enzyme abundance and functionality. BSD2 is a Rubisco-specific assembly chaperone that assembles Rubisco large and small subunits into the functional holoenzyme. Overexpressing *BSD2* increased both the content and activation state of Rubisco in maize and rice (Tominaga et al., 2020). In a related study, Whitney et al. (2015) introduced the *RbcL* gene from Arabidopsis (*Arabidopsis thaliana*) into the chloroplasts of tobacco plants along with the chaperone gene *AtRAF1*. This co-expression increased the biogenesis of hybrid Rubisco 2 to 3-fold, resulting in enhanced CO_2_ assimilation in leaves and increased plant growth. These findings demonstrate that chaperone compatibility is a critical determinant of successful recombinant Rubisco engineering (Orr et al., 2023; Prywes et al., 2023). RAF1 specifically binds to and stabilizes newly translated Rubisco large subunits to prevent them from aggregating, whereas RAF2 is a fusion chaperone that facilitates the assembly of large and small subunits into holoenzymes (Huang et al., 2020). However, the exact molecular details of RAF1, RAF2, and BSD2 function remain largely unknown (Whitney et al., 2015). It is premature to assign roles to these proteins and assume that they interact with Rubisco in chloroplasts, warranting further studies.

### 2.3 Engineering Rubisco beyond native constraints: heterologous Rubiscos, directed evolution, and assembly optimization

To circumvent the intrinsic kinetic limitations of plant Rubisco, researchers have turned to Rubisco isoforms from bacteria and algae. For instance, a native tobacco *RbcL* gene was genetically replaced with *Halothiobacillus neapolitanus* Rubisco (HnRubisco) large and small subunit genes in chloroplasts, leading to a two-fold higher carboxylation rate than plant Rubisco under elevated CO_2_ conditions (Chen et al., 2023). In parallel, replacing plant Rubisco with Rubiscos from red algae, with greater CO_2_ specificity, has been investigated. However, the complex folding requirements of these Rubiscos require the co-expression of genes encoding specialized chaperones and assembly factors (Hauser et al., 2015; Whitney et al., 2015). **Table 1** summarizes representative forms of Rubisco from plants, algae, cyanobacteria, archaea, and engineered hybrids.

**Table 1:** Rubisco isoforms with potential applications for engineering plant photosynthesis.

Rubisco type	Organism	Kinetics (k_cat__,c_ s^-^¹; K_c_ µM; S*_c/o_*)	Feasibility for specific applications	References
Form IA	*Synechococcus elongatus*	9.8; 152; 50.3	High: CCM, successful chloroplastic expression with RAF1 and RbcX	(Shih et al., 2016)
	*Hydrogenovibrio marinus* (*CbbL1S1*)	0.9; NA; 30.9	Moderate: non-carboxysomal	(Hayashi et al., 1998)
	*Rhodobacter capsulatus*	2.5; 22.1; 25.9	Moderate: non-photosynthetic potential	(Horken and Tabita, 1999)
	*Halothiobacillus neapolitanus* proteobacterium	2–5; 20; NA	High	(Chen et al., 2023)
Form IB	*Arabidopsis thaliana*, *Nicotiana tabacum*,	3.0; 9.8; 80	Very high: natural form in C_3_ crops; target of subunit substitutions	(Whitney et al., 2015)
	*Oryza sativa* ssp*. indica*	2.2; 7.0; 101	Very high: C_3_ rice; engineering target	(Orr et al., 2016)
	*Sorghum bicolor*	5.8; 22.9; NA	High: C_4_ crop	(Sharwood et al., 2016)
	*Zea mays*	5.5; 18.9; 88	High: C_4_ crop	(Sharwood et al., 2016)
	*Chlamydomonas reinhardtii*	1.8; 30; 64	High: green alga	(Zhu and Spreitzer, 1994)
	*Scenedesmus obliquus*	38.0; 63; NA	High k_cat_; potential in synthetic biology	(Savir et al., 2010)
Form IC	*Cupriavidus necator*	2.1; 50.2; 74	Moderate: limited engineering potential	(Lee et al., 1991)
	*Bradyrhizobium japonicum*	2.2; 50.2; 74.8	Moderate: nitrogen-fixing species	(Horken and Tabita, 1999)
	*Xanthobacter flavus*	1.4; 76.1; 44.4	Moderate: proteobacteria	(Horken and Tabita, 1999)
Form ID	Red microalgae *Galdieria sulphuraria*	1.2; 3.3; 166	High: high specificity and thermostable	(Whitney et al., 2001)
	Red macroalgae *Griffithsia monilis*	2.6; 9.3; 167	Moderate	(Whitney et al., 2001)
	*Palmaria decipiens*	2.4; 17.4; NA	Red macroalga	(Iñiguez et al., 2018)
	*Cyanidium caldarium*	1.3; 6.7; 224.6	Red microalga	(Uemura et al., 1997)
Form II	*Rhodospirillum rubrum*	8–10; 45–60; 30–35	Low: non-chloroplast assembly	(Christeller and Laing, 1978)
Form III	*Archaeoglobus fulgidus*	2–3; 50–60; 20–25	Minimal: non-photosynthetic AMP pathway	(Finn and Tabita, 2003)
Hornwort-type (IB variant)	*Anthoceros agrestis*	3–10; 30; 70	High: compatible with pyrenoid CO_2_ concentrating mechanism	(Oh et al., 2024)
Synthetic hybrid	Cyanobacteria–tobacco		High: CCM approach	(Orr et al., 2020)

Directed evolution approaches have also been used to improve the catalytic efficiency and substrate specificity of Rubisco using microbial production systems. In nature, Rubisco has four distinct isoforms (I–IV) categorized by their quaternary structure, chaperones used for assembly, and origin. Form I Rubiscos dominate photosynthetic CO_2_ fixation in crops and are the primary targets of engineering, whereas Form II–IV Rubiscos remain underexplored due to structural incompatibility with plant plastids. In *E. coli*, variants of cyanobacterial Rubisco (Form I) such as those harboring a F345I substitution displayed improved folding efficiency and lower *K_m_* values for RuBP (Mueller-Cajar and Whitney, 2008). Similarly, Rubisco variants from archaea carrying E138V and K332E substitutions showed more than three-fold higher catalytic efficiency and enhanced photosynthesis compared to the intact version when introduced into tobacco (Wilson et al., 2016). *RCA* has also been subjected to mutagenesis, generating variants encoding an enzyme with enhanced redox stability and ATP sensitivity that supports Rubisco performance under stress conditions (Scafaro et al., 2018). Despite promising improvements in the kinetic parameters of Rubisco in microbial systems, the assembly of these single gene–derived variants into plants remains constrained by incompatibility with the endogenous photosynthetic machinery, chaperone requirements, and stromal ATP/pH regulation. Similarly, certain Rubisco isoforms show lower CO_2_ specificity (*K_c_*) when overexpressed in plants despite exhibiting higher catalytic turnover (*k_cat_*). Additionally, the modified Rubisco variants are often incompatible with native plant RCAs and associated subunits or depend on additional chaperones. Thus, Rubisco engineering must be considered together with that of the associated assembly machinery and the surrounding stromal environment to enhance C*_i_* concentrations (Occhialini et al., 2016).

### 2.4 Carbon-concentrating mechanisms

To enhance CO_2_ availability and minimize photorespiration, biophysical CCMs have been engineered into C_3_ crops. This strategy addresses a fundamental limitation of Rubisco: it catalyzes both carboxylation and oxygenation reactions at the same active site. Increasing the CO_2_ concentration around Rubisco shifts its preference toward carboxylation over wasteful oxygenation (Betti et al., 2016; Hagemann and Bauwe, 2016; Nolke et al., 2019). Unlike C_3_ plants, in which the oxygenase activity of Rubisco leads to considerable resource losses through photorespiration, C_4_ and CAM plants, as well as cyanobacteria and algae, have evolved CCMs that concentrate CO_2_ around Rubisco, thereby enhancing its carboxylase function.

The CCM in cyanobacteria relies on the formation of carboxysomes; these protein-based microcompartments encapsulate Rubisco together with carbonic anhydrase (Trettel et al., 2024). Modeling by McGrath and Long (2014) predicted that introducing a fully functional cyanobacterial CCM into C_3_ crops could improve photosynthetic efficiency and yield by up to 60%, largely by boosting CO_2_ concentrations without increasing the demand for leaf nitrogen (McGrath and Long, 2014). Introducing the cyanobacterial CCM into plants would require the coordinated expression of genes encoding 16–18 novel components spanning shell proteins, enzymes, and uptake systems beyond native Rubisco.

Fang et al (Fang et al., 2018). created an IPTG-inducible synthetic *CCM* cassette hosting 12 genes from *Synechococcus elongatus* PCC7942 encoding shell proteins (CcmK2/3/4, CcmO/L/P/N), Rubisco linker (CcmM), Rubisco (rbcL/S), chaperone (RbcX), and carbonic anhydrase (CcaA). When expressed in *E. coli*, this generated slightly larger, irregularly shaped carboxysome-like structures, suggesting that optimization of the ratios of these components is required. A foundational study by Lin et al. (2014) demonstrated the feasibility of generating transplastomic plants: Directly replacing native tobacco *rbcL* with homologous genes from *Synechococcus elongatus* PCC7942 (*RbcL*, *RbcS*, *RbcX*, or *CcmM35*) in the tobacco plastid genome via homologous recombination achieved correct chloroplast targeting and holoenzyme assembly (Lin et al., 2014). Complementary efforts have focused on introducing cyanobacterial inorganic carbon uptake systems; bicarbonate transporter genes such as *BicA* and *SbtA* have been successfully expressed in Arabidopsis, leading to inorganic carbon accumulation analogous to that produced by cyanobacterial C*_i_* transport mechanisms (Rolland et al., 2016; Uehara et al., 2016). As a further step toward CCM reconstitution, Long et al. (2018) expressed genes encoding minimal carboxysome shell proteins (CsoS1A and CsoS2) in tobacco chloroplasts, leading to the self-assembly of partial carboxysome-like structures (Long et al., 2018). Although these structures were not fully functional, as they lacked the full set of proteins needed to concentrate CO_2_ efficiently, their formation marked a major milestone in demonstrating the feasibility of carboxysome biogenesis in land plants. However, reconstituting a fully functional CCM requires the coordinated installation of C*i* transport systems, carboxysome assembly, control of stromal pH, and suppression of stromal carbonic anhydrase, making this one of the most complex synthetic biology challenges in plants (Hennacy and Jonikas, 2020).

A distinct biophysical CCM operates in the green alga Chlamydomonas, whose Rubisco is concentrated into a specialized structure known as the pyrenoid. This condensate forms through interactions between Rubisco and the linker protein EPYC1, generating a dense matrix that helps increase CO_2_ concentrations. Co-expressing *EPYC1* with an *RBCS* gene from Chlamydomonas promoted the formation of proto-pyrenoid structures in transgenic Arabidopsis plants, demonstrating the cross-kingdom compatibility of condensate formation (Atkinson et al., 2019). Fei et al. (2022) identified essential components required to reconstruct a minimal functional algal CCM by examining all Chlamydomonas pyrenoid-based CCM (PCCM) deficient mutants (Fei et al., 2022). These components include a physical barrier (such as a starch sheath) to limit CO_2_ leakage outside the pyrenoid, thylakoid tubules that penetrate the pyrenoid to deliver CO_2_ to Rubisco via the lumen, and proper enzyme placement to minimize wasteful cycling between CO_2_ and HCO_3_^−^. The active pumping of HCO_3_^−^ into the stroma enhances CCM performance under low CO_2_ conditions. Computational modeling suggested that implementing such a system could potentially triple the CO_2_ assimilation rate, with an estimated energetic cost of only ~1.3 ATP per CO_2_ fixed, unlike the 3 ATPs/CO_2_ required by the C_3_ Calvin cycle.

In parallel with these synthetic biophysical CCM approaches, considerable progress has been made toward introducing C_4_ photosynthesis into C_3_ crops. C_4_ metabolism concentrates CO_2_ around Rubisco by spatially segregating initial carbon fixation and decarboxylation between mesophyll and bundle sheath cells (Langdale, 2011). In the past, converting the C_3_ crop rice into a C_4_ plant typically involved stacking genes by repeated crossing or retransformation, achieving high *in vitro* enzyme activity but failing to form C_4_ compounds *in vivo* due to a lack of cell specificity, incorrect subcellular targeting, and inadequate metabolite transport ability (Taniguchi et al., 2008; Miyao et al., 2011). Advanced engineering efforts have combined the heterologous expression of genes encoding key C_4_ enzymes, including phosphoenolpyruvate carboxylase (PEPC), NADP-malic enzyme (NADP-ME), and pyruvate phosphate dikinase (PPDK), together with attempts to modify leaf anatomy to approximate Kranz-like organization (Jafarpour and Nulit, 2011; Langdale, 2011). Although the installation of a complete C_4_ pathway remains a major challenge, the partial implementation of C_4_ traits in rice has already produced measurable improvements in photosynthetic performance, particularly under high-light and drought conditions (Li et al., 2020; Ermakova et al., 2021a).

### 2.5 Adding the alternative C_2_ photosynthetic pathway to crops

To minimize photorespiratory CO_2_ loss, some plant lineages have evolved C_2_ photosynthesis. This photorespiratory CO_2_-concentrating mechanism represents an intermediate evolutionary state between C_3_ and C_4_ photosynthesis (Walsh et al., 2023). Here, the two-carbon molecule glycine is shuttled between cells during photorespiration (Raghavendra and Sage, 2010; Lundgren, 2020). In general, glycine produced in the mesophyll cells of C_3_ plants during photorespiration is decarboxylated by mesophyll glycine decarboxylase (GDC) to release CO_2_, which is lost by diffusion (Kennedy et al., 1980). By contrast, C_2_ plants have a characteristic Kranz-like leaf ultrastructure, with relatively enlarged bundle sheath cells hosting Rubisco and abundant mitochondria (Vogan et al., 2007). The GDC activity required to complete photorespiration in C_2_ plants is primarily restricted to mitochondria localized to the inner walls of bundle sheath cells (Hylton et al., 1988; Morgan et al., 1993; Khoshravesh et al., 2016; Sage, 2017). Therefore, the glycine formed in mesophyll cells must be shuttled to bundle sheath cells where GDC releases CO_2_ and concentrates it closer to Rubisco, increasing leaf CO_2_ levels by approximately three-fold compared to C_3_ plants, thus increasing the carboxylation efficiency of Rubisco (Monson and Rawsthorne, 2000; Monson, 2003; Keerberg et al., 2014). Although C_2_ plants lack the complete CO_2_-concentrating system of C_4_ plants, they exhibit less photorespiratory CO_2_ loss than C_3_ plants. Although these plants are not widely prevalent in nature, they possess considerable evolutionary significance and have attracted interest for potential engineering into C_3_ crops (Lundgren, 2020).

Although C_3_ photosynthesis predominates in major crop species, photorespiration under elevated temperature and low CO_2_ conditions causes substantial losses of assimilated carbon, nitrogen, ATP, and reducing equivalents. While C_4_ photosynthesis effectively minimizes these losses through a physiochemical CCM, its transfer into C_3_ crops is highly challenging. This is primarily due to the requirement for extensive anatomical modifications (*e.g.*, Kranz anatomy) and the coordinated metabolite transport and spatial functioning of enzymes (Cui, 2021). Alternatively, C_2_ species experience relatively low net carbon loss through photorespiration. C_3_ plants host all the C_2_-like enzymes required for photorespiratory metabolism (Lundgren, 2020). Therefore, engineering a C_2_ pathway into C_3_ crops could offer a meaningful opportunity to improve crop productivity. However, the successful implementation of this strategy would require spatial restriction of glycine decarboxylase (GDC) gene expression to bundle sheath cells. *GLDP* encodes the P-protein (pyridoxal phosphate–dependent decarboxylase subunit) of the GDC complex. The bundle sheath-specific localization of this subunit is essential for redirecting glycine to bundle sheath cells for decarboxylation, thereby concentrating CO_2_ closer to Rubisco for recapture (Dickinson et al., 2025).

The M-box is a cis-regulatory element in the Arabidopsis *GLDP1* promoter that drives its expression in mesophyll cells. The deletion or loss of the M-box abolished *GLDP1* expression in mesophyll cells and promoted bundle sheath–localized *GLDP* expression (Adwy et al., 2019). In C_3_ plants, *GLDP* is under strict regulation by two components: an M-box-dependent activation system in mesophyll cells and a MYC–MYB transcription factor-mediated system in bundle sheath cells. During the evolution of C_2_ photosynthesis, the loss of M-box regulatory input and retention of the MYC–MYB program restricted *GLDP* expression to bundle sheath cells, which is required for generating photorespiratory CO_2_ concentrations (Dickinson et al., 2025). These findings support the feasibility of C_2_ engineering via CRISPR-based promoter editing to spatially express photorespiratory genes. However, successful C_2_ engineering also requires coordinated improvements in metabolite transport, nitrogen allocation, mitochondrial positioning, and plasmodesmatal connectivity to support increased flux.

A model simulation performed by Bellasio and Farquhar (2019) on C_3_ rice with C_2_ enzymes predicted not only an increased rate of CO_2_ assimilation under ambient environmental conditions, but also under high temperature (>35 °C), high light (>700 µmol m^-^² s^-^¹), and low CO_2_ conditions (<400 µmol mol^-^¹). Building on these findings, it would be useful to test this regulatory logic in agronomically important crops in the future.

### 2.6 Complementary strategies for enhanced carboxylation

**Figure 2:**
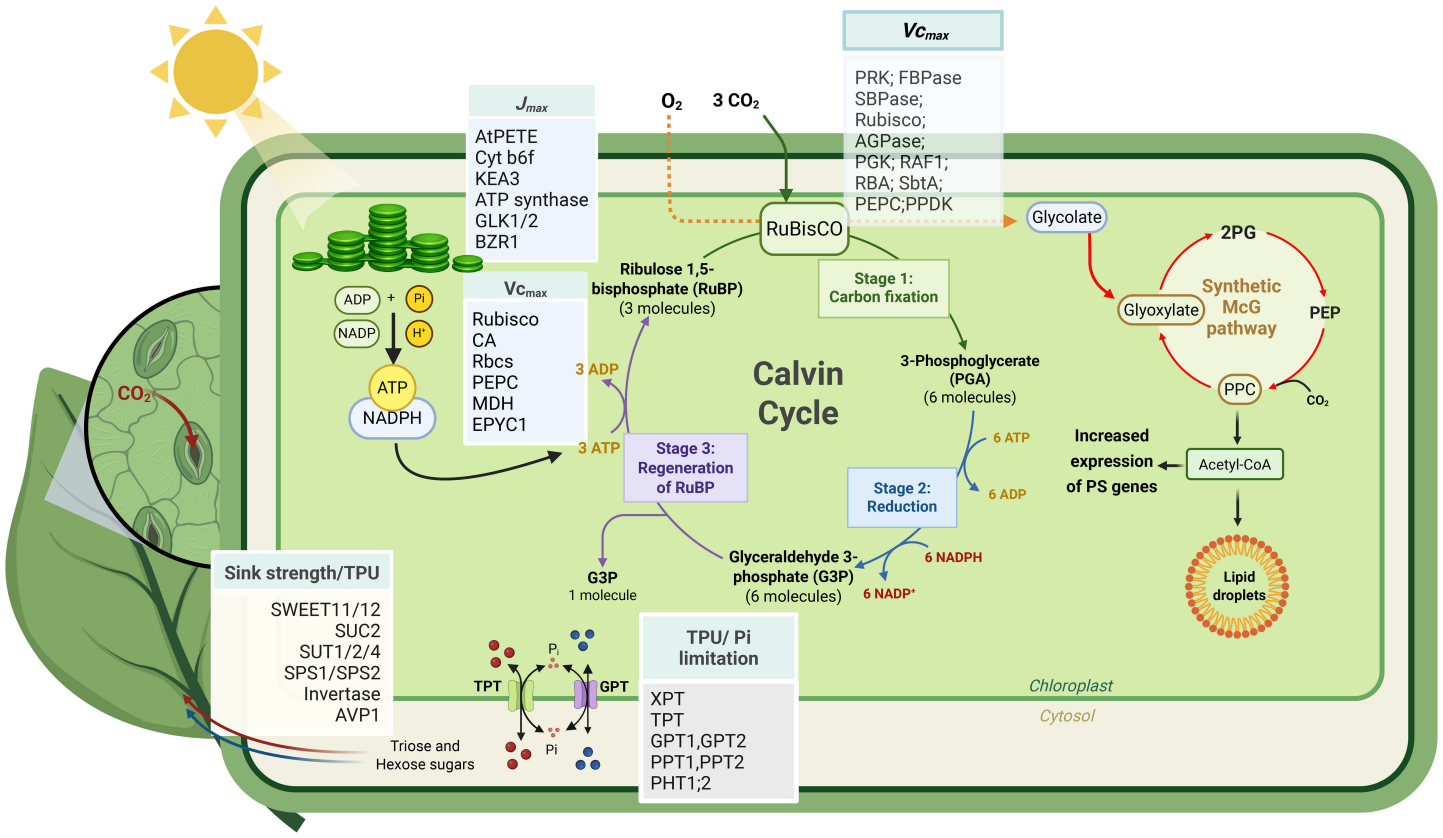
Schematic representation of photosynthetic carbon assimilation and proven biotenchnological targets capable of alleviating biochemical limitation when employed individually or in combination. Overall, the incident photons drives the photosynthetic electron chain in thylakoid membrane to generate reducing power NADPH and ATP to power Calvin-Benson cycle in the chloroplast. The increased expression of photosynthesis genes and components such as AtPETE, Cyt b_6_f, KEA3, ATP synthase, GLK1/2, and BZR1 enhances light harvesting, electron transport rate (*Jmax*) and photoprotection, thereby improving PS efficiency. The major CBB enzymes and Rubisco biogenesis factors gene expression (PRK, FBPase, SBPase, PGK, AGPase, RAF1, RBA, SbtA, PEPC, and PPDK) coupled with CCMs interventions (Rubisco, CA, RCA, RbcS, PEPC, MDH, and EPYC1) enhances Rubisco carboxylation capacity (VC*_max_*) by increasing CO_2_ availability. The synthetic pathway introduction of glycolate metabolism (McG- methylglyoxal; highlighted with red arrows), suppress photorespiratory carbon loss by recycling it for enhanced acetyl-Co-A production or lipid biosynthesis. At the chloroplast envelope, metabolite transporters (XPT, TPT, GPT1, GPT2, PPT1, PPT2, and PHT1:2) regulate triose-phosphate export and inorganic phosphate (Pi) recycling. Enhancing sink strength and improved triose- phosphate utilization (TPU) are achieved by increased expression or activity of sugar transporters and sucrose-metabolism components such as SWEET11/12, SUC2, SUT1/2/4, SPS1/SPS2, AVP1and invertase enzymes. Together, these modifications promote efficient sugar loading, transport, and allocation to developing sink tissues, thereby preventing feedback inhibition in source leaves and sustaining high rates of photosynthetic carbon assimilation. AtPETE, Arabidopsis plastocyanin; Cyt b f, cytochrome bef complex; KEA3, K+ efflux antiporter 3; GLK1/2, GOLDEN2-LIKE1/2; BZR1, BRASSINAZOLE-RESISTANT 1; Rubisco, ribulose-1,5-bisphosphate carboxylase/oxygenase; CA, carbonic anhydrase; RCA, Rubisco activase; RbcS, Rubisco small subunit; PEPC, phosphoenolpyruvate carboxylase; MDH, malate dehydrogenase; EPYC1, essential pyrenoid component 1; PRK, phosphoribulokinase; FBPase, fructose-1,6-bisphosphatase; SBPase, sedoheptulose-1,7-bisphosphatase; PGK, phosphoglycerate kinase; AGPase, ADP-glucose pyrophosphorylase; RAF1, Rubisco accumulation factor 1; RBA, Rubisco biogenesis/activation factor (as defined in text); SbtA, sodium bicarbonate transporter A; PPDK, pyruvate, phosphate dikinase; XPT, xylulose 5-phosphate/phosphate translocator; TPT, triose-phosphate/phosphate translocator; GPT1/2, glucose-6-phosphate/phosphate translocator 1/2; PPT1/2, phosphoenolpyruvate/phosphate translocator 1/2; PHT1:2, phosphate transporter 1;2; SWEET11/12, Sugars Will Eventually be Exported Transporter 11/12; SUC2, sucrose-proton symporter 2; SUT1/2/4, sucrose transporters 1/2/4; SPS1/2, sucrose-phosphate synthase 1/2; AVP1, Arabidopsis vacuolar H-pyrophosphatase 1; McG, modified glycolate/photorespiratory cycle; TPU, triose-phosphate utilization; PS, photosynthesis. This image created in BioRender 54e39i3.

Although optimizing Rubisco and incorporating CCMs are promising directions, several other strategies have shown potential for enhancing photosynthetic carbon assimilation. For example, synthetic photorespiratory bypasses can help minimize the carbon and energy losses associated with endogenous photorespiration. Over 30 synthetic CO_2_-fixing pathways have been designed *in silico* with computationally improved thermodynamics and kinetics properties compared to the existing CCB cycle, although most await *in vivo* testing (Bar-Even et al., 2010; Luo et al., 2022). A landmark example was the successful construction of the malyl-CoA–glycerate (McG) cycle in Arabidopsis (Lu et al., 2025). The McG cycle is a synthetic carbon-fixation pathway designed to minimize carbon loss from both the CBB cycle and photorespiration (**Figure 2**). Transgenic McG plants exhibited 2–3-fold increases in biomass. The enhanced PSI and PSII efficiency, higher abundance of electron transport and ATP synthase components, and elevated cytokinin levels have collectively resulted in more and larger leaves and greater seed yield. Mechanistically, the McG cycle employs phosphoenolpyruvate carboxylase (PEPC) to convert either glycolate or 3-phosphoglycerate (3PG) into acetyl-CoA, thereby fixing one additional carbon atom (from 3PGA) or avoiding CO_2_ release entirely (from glycolate).

This successful example builds on earlier chloroplast-targeted photorespiratory bypass methods that also aimed to recycle glycolate more efficiently. For example, Maier et al. (2012) introduced a chloroplast-localized glycolate catabolic cycle into Arabidopsis, generating GMK plants. These GMK plants harbored glycolate oxidase, which initiates glycolate oxidation; malate synthase channels, allowing the resulting carbon to function as a useful metabolic intermediate; and catalase, which detoxifies the H_2_O_2_ produced by glycolate oxidase. This combination improved photosynthetic performance and biomass accumulation, likely by reducing photorespiratory carbon loss and altering the glycine/serine balance.

Shen et al. (2019) introduced a chloroplast photorespiratory bypass (GOC) into rice by constitutively expressing genes encoding glycolate oxidase 3 (*OsGLO3*), oxalate oxidase 3 (*OsOXO3*), and catalase (*OsCATC*). The strategy enhanced photosynthetic efficiency and biomass but impaired seed-setting ability. Perhaps the continuous glycolate oxidase activity led the accumulation of high levels of H_2_O_2_ inside the plastid, potentially disrupting carbon allocation and reproductive development (Fahnenstich et al., 2008). A modified version of the GOC bypass, the MGA bypass, was developed in rice (Xu et al. (2023). In this study, *OsGLO1* expression was controlled by the light-inducible rice Rubisco small subunit promoter (pRbcS), allowing dynamic regulation under fluctuating light conditions, whereas the *Cucurbita maxima* malate synthase gene *CmMS* and the rice ascorbate peroxidase 7 (*OsAPX7*) gene were constitutively overexpressed. Compared with the original GOC bypass, the MGA bypass achieved a moderate but sustained increase in photosynthesis and improved yield without affecting seed-setting ability.

In the past, South et al. (2019) engineered a chloroplast-targeted bypass in tobacco by introducing a synthetic pathway consisting of Chlamydomonas glycolate dehydrogenase (CrGDH) and winter squash (*Cucurbita maxima*) malate synthase (MS) while simultaneously suppressing the endogenous glycolate transporter plastidic glycolate/glycerate transporter 1 (PLGG1) through RNA interference of its encoding transcripts. This modification led to complete glycolate metabolism within chloroplasts, effectively bypassing the native, energy-intensive pathway. Similarly, the Kebeish group (Kebeish et al., 2007) introduced bacterial-based bypass in Arabidopsis using the *E. coli* glycolate dehydrogenase (EcGDH), glyoxylate carboligase (EcGCL), and tartronate semialdehyde reductase (EcTSR), which South et al. (2019) subsequently combined with *PLGG1* suppression to retain glycolate in the chloroplast. Notably, several metabolic complications arise from these approaches that require corrective strategies. GDH-based pathways generate free glyoxylate, thus creating an NADPH imbalance that can potentially impair the CBB cycle. GO-based approaches generate H_2_O_2_, requiring the co-expression of *CAT* to protect subcellular membranes and photosystem II (PSII) stability (Trudeau et al., 2018; Shen et al., 2019; South et al., 2019). In addition, bypass pathways can alter the redox balance in chloroplasts, nitrogen reassimilation, and reactive oxygen species metabolism, all requiring careful metabolic tuning. The success of the McG and glycolate catabolic pathways demonstrates the transformative potential of synthetic carbon-fixation strategies to work around the inherent limitations of the CBB cycle.

Structural reconfiguration within chloroplasts represents another promising avenue for enhancing photosynthetic efficiency. Overexpressing *GOLDEN2-LIKE* (*GLK*) transcription factor improved thylakoid architecture, photosystem organization, and electron transport efficiency (Waters et al., 2009; Tokumaru et al., 2017). Beyond biochemical and structural modifications, improving mesophyll conductance (g_m_) can significantly improve CO_2_ diffusion to the chloroplast. Genetic strategies such as overexpressing the aquaporin gene *PIP1;2* (*PLASMA MEMBRANE INTRINSIC PROTEIN 1;2*) or lowering cell-wall resistance increased CO_2_ permeability, thereby improving the photosynthetic performance of the C_4_ species *Setaria viridi*s (Ermakova et al., 2021b; Ding et al., 2024). However, these strategies come with side effects, including higher trans-cuticular water loss and diminished drought tolerance, lower mechanical strength stemming from thinner cell walls, and greater susceptibility to pathogen invasion (Flexas et al., 2021). As a step forward in g_m_ engineering, constitutive overexpression of *COTTON GOLGI-RELATED 3* (*AtCGR3*, encoding a pectin methyltransferase) in tobacco showed promising results, with an 8% higher photosynthetic rate due to thinner cell walls (Salesse-Smith et al., 2024). This finding offers potential alternatives to enhance the CO_2_ supply while limiting the drawbacks posed by altering g_m_. **Table 2** lists the various other strategies used to increase the carboxylation capacity of Rubisco.

**Table 2:** Biotechnological approaches to enhance the carboxylation capacity of Rubisco to improve photosynthetic performance.

Organism	Engineering strategy	Observed photosynthetic & yield outcome	References
Tobacco	Overexpressing the Arabidopsis Rubisco chaperone gene *RAF1* and Rubisco subunit to enhance assembly and activation	Increased Rubisco content and activity; increased the photosynthetic rate	(Whitney et al., 2015)
Maize	Engineering of Rubisco activase isoforms (*α* and *β*)	Enhanced Rubisco activation and photosynthetic rate	(Yin et al., 2022)
Wheat	Increasing SBPase activity by overexpression	Enhanced photosynthetic rate, biomass, and yield	(Driever et al., 2017)
Potato	Expression of the glycolate dehydrogenase gene (*GDH*)	Increased CO_2_ assimilation rate and biomass accumulation	(Nölke et al., 2014)
Arabidopsis	Overexpression of *SaPEPC1*	Increased photosynthetic rate under intense light	(Li et al., 2025)
Cotton	Overexpression of *RCA* and *AVP1*	Cotton plants showed a 6.5-fold increase in net photosynthetic rates and higher *Vc_max_* rates under multiple stress conditions	(Smith et al., 2023)
Arabidopsis	Co-overexpression of *AVP1* and *OsSIZ1* for sustained photoassimilation under stress conditions	Improved plant performance under multiple stress conditions	(Esmaeili et al., 2019)
Tomato	Overexpression of *BZR1* to trigger CBB enzymes	Enhanced accumulation of the CBB enzymes FBA1, RCA1, and FBP5 and enhanced *Vc_max_*and *J_max_*	(Yin et al., 2022)
Rice	Increasing Rubisco small subunit (*RbcS*) expression to enhance holoenzyme formation	Increased Rubisco content and photosynthetic rate	(Suzuki et al., 2007)
Arabidopsis	Increasing the thermal stability of RCA to maintain activity at high temperatures	Increased photosynthetic rate under heat stress	(Kurek et al., 2007)
Rice	Co-overexpression of *Rubisco* (*Rb*c*S*) and *RCA* under heat stress	Increased photosynthetic rate and yield	(Qu et al., 2021)
Arabidopsis	Overexpression of a C_4_-specific carbonic anhydrase gene for PEPC	Increased carboxylation and starch and sucrose contents	(Kandoi et al., 2022)
Rice, maize, sorghum, tobacco	Overexpression of *RbcS* for enhanced Rubisco content	Increased Rubisco content (125–130%) and yield potential under nitrogen-sufficient conditions	(Chen et al., 2023)
Rice	Overexpression of *OsCGA1* for chloroplast biogenesis	Induced photosynthetic gene expression	(Lee et al., 2021)
Arabidopsis	Overexpression of *PIP1;2* (mesophyll-expressed aquaporin gene)	Increased CO_2_ diffusion and assimilation efficiency	(Kromdijk et al., 2020)

## 3 Biochemical Limitations of RuBP Regeneration

RuBP is a five-carbon CO_2_ acceptor molecule that is carboxylated by Rubisco in the CBB cycle (Farquhar et al., 1980). When Rubisco-mediated carboxylation no longer limits CO_2_ assimilation, RuBP regeneration becomes the primary determinant of photosynthetic capacity. In each CBB cycle, RuBP fixes an incoming CO_2_ molecule to 3PGA, some of which is utilized in the regeneration of RuBP. Therefore, when the capacity to regenerate RuBP is insufficient to match the carboxylation demands of Rubisco, photosynthesis is limited. The regeneration process begins with the reduction of 3-phosphoglycerate (3PGA) to triose phosphates (glyceraldehyde-3-phosphate-GAP and dihydroxyacetone phosphate-DHAP) via ATP and NADPH-dependent reactions catalyzed by phosphoglycerate kinase and glyceraldehyde-3-phosphate dehydrogenase (Sage, 1990; Bernacchi et al., 2003). The resulting triose phosphates enter a sequence of reactions catalyzed by aldolase, transketolase, FBPase, and SBPase to yield ribose-5-phosphate (Ru5P). Finally, phosphoribulokinase (PRK) phosphorylates Ru5P in an ATP-dependent reaction to regenerate RuBP (Foyer et al., 2012). These enzymes take part in the regenerative phase of the CBB cycle and are also subject to light- and redox-dependent regulation. Although NADPH is required only during the reduction of 1,3-bisphosphoglycerate to GAP, it indirectly supports RuBP regeneration by providing reduced carbons during the reductive phase of the CBB cycle. Therefore, due to the close association of light-driven electron transport in the thylakoid, which yields ATP and NADPH to power the CBB cycle, maximum electron transport rate (*J_max_*) is one of the primary determinants influencing RuBP regeneration (Farquhar et al., 1980; Walker et al., 2014). Thus, RuBP regeneration represents a critical integration point among carboxylation, electron transport, and carbon metabolism.

However, environmental constraints, including low CO_2_ levels, drought, high light, temperature stress, and phosphorus deficiency, can impair RuBP regeneration and shift photosynthesis from a process limited by Rubisco to a process limited by RuBP regeneration. This transition is represented in the FvCB model by the *J_max_*-limited phase, where RuBP regeneration becomes the primary limitation to photosynthesis (**Figure 1**).

### 3.1 Engineering photosynthetic electron transport for improved RuBP regeneration

Photosynthesis functions via a coordinated metabolic network. Constraints in one step obscure downstream processes and reduce overall photosynthetic flux. In many crops, the electron transport chain appears to be suboptimal under prevailing environmental conditions (Gu, 2023). Photosynthetic electron transport capacity is positively correlated with cytochrome contents in leaves across plant species, which was shown to affect overall plant growth (Evans and Seemann, 1989; Chida et al., 2007; Simkin et al., 2017b; Ermakova et al., 2019). Genetic evidence supports this relationship: antisense-mediated suppression of the gene encoding Rieske Fe-S protein, a core component of the cytochrome b_6_f complex (composed of Cyt *f* or PetA, Cyt *b_6_* or PetB, Rieske Fe-S or PetC, and Subunit IV or PetD), led to a proportional drop in the electron transport rate (Price et al., 1995; Yamori et al., 2011). During active photosynthesis, Cyt b_6_f regulates the electron transport rate by channeling electrons to PSI and protons to the thylakoid lumen for ATP biosynthesis. Conversely, when CO_2_ fixation slows, acidification of the lumen suppresses electron flow to PSI, resulting in over-reduction and the activation of NPQ. Ectopic expression of *Bracchypodium distachyon* Rieske Fe-S subunit gene (*PetC*) in Sorghum and *S. viridis* (C_4_ plants) enhanced CO_2_ assimilation under elevated CO_2_ conditions, respectively (Ermakova et al., 2019, 2023). Overexpressing this gene in tobacco resulted in a >40% increase in Cyt b_6_f abundance and faster photosynthetic induction under fluctuating light conditions but no increase in steady-state CO_2_ assimilation (Heyno et al., 2022). Introducing the algal cytochrome c6 into the photosynthetic electron transport chain of Arabidopsis (Chida et al., 2007) and tobacco (Yadav et al., 2018) enhanced the photosynthetic rate, chlorophyll contents, photosynthetic pigment ratios, and water-use efficiency. These findings highlight the importance of the cytochrome complex in sustaining higher photosynthetic rates and biomass accumulation. However, whether increased cytochrome abundance can consistently enhance steady-state CO_2_ assimilation and translate into yield gains across diverse C_3_ species remains unresolved. Additionally, cytochrome-mediated enhanced electron transport may become uncoupled from carbon-fixation capacity under metabolic or environmental limitations. Therefore, increasing the abundance of the cytochrome complex might be more effective when combined with Rubisco optimization and the coordinated enhancement of CBB cycle enzymes.

An additional yet relatively underexplored limitation of photosynthetic gains arises from the slow activation of photochemical reactions during transitions between light and shade. This phenomenon occurs due to the delayed oxidation of plastoquinone (PQ) and the slow activation of the Cyt b_6_f complex and PSI acceptors. Under field conditions, such a delay in induction can result in substantial losses in daily carbon assimilation, reaching up to 20% in crops such as wheat and rice (Taylor and Long, 2017; Leister, 2023). Collectively, these observations emphasize the need for strategies that enhance both the structural integrity and regulatory responsiveness of the photosynthetic electron transport chain, which indirectly affect RuBP regeneration.

### 3.2 Coordinated enhancement of the CBB cycle and electron transport rate

Although increasing the carboxylation capacity of Rubisco (Vc*_max_*) has long been a central strategy for enhancing photosynthesis, sustained gains in carbon assimilation have also been observed with increases in RuBP regeneration (J*_max_*) (Evans, 2013). The J*_max_* to Vc*_max_* ratio represents a critical axis of resource allocation in photosynthesis, balancing CO_2_ fixation capacity with RuBP regeneration. Substantial variation in this ratio has been reported across species and environments, reflecting both external drivers such as temperature, atmospheric CO_2_, and soil nutrient levels (largely nitrogen and phosphorus) and intrinsic plant factors such as the stage of leaf development, canopy position, and structural traits such as specific leaf area (Walker et al., 2014). When Vc*_max_* exceeds J*_max_*, photosynthesis becomes limited by electron transport and RuBP regeneration, whereas an excessively high J*_max_* relative to Vc*_max_* shifts the limitation toward Rubisco activity. Optimizing the Vc*_max_* to J*_max_* ratio is therefore critical but context dependent. Reported J_max_/Vc_max_ ratios in the tropics range from 1.08 to 2.24. Cooler environments tend to lead to higher ratios, reflecting a greater investment in RuBP regeneration capacity under these conditions (Song et al., 2021). Because J*max* largely reflects the biochemical capacity for RuBP regeneration, enhancing its efficiency through the coordinated manipulation of key CBB cycle enzymes has emerged as a major strategy to improve photosynthetic carbon assimilation (Raines, 2022).

The regeneration of RuBP requires the coordinated action of eight CCB cycle enzymes (**Figure 2**) that convert glyceraldehyde 3-phosphate (GAP) into RuBP, using ATP supplied by photochemical reactions (Raines, 2022). Antisense suppression studies have helped identify CBB cycle enzymes that exert strong control over photosynthetic flux. Among these, SBPase and fructose-1,6-bisphosphate aldolase (FBPA) consistently emerge as key regulators (Stitt and Schulze, 1994; Raines, 2003). Overexpression of *SBPase* in Arabidopsis, tobacco, wheat, rice, and tomato (*Solanum lycopersicum*) led to significant increases in photosynthetic capacity and biomass under both controlled and field conditions (Lefebvre et al., 2005; Simkin et al., 2015; Driever et al., 2017; Suzuki et al., 2019). Increased SBPase activity also conferred enhanced chilling tolerance and photosynthetic performance in tomato (Ding et al., 2017).

Similarly, overexpressing *FBPA*, encoding a key Calvin cycle enzyme that cleaves fructose-1,6-bisphosphate into GAP and dihydroxyacetone phosphate, improved photosynthesis and biomass production in tobacco (Uematsu et al., 2012; Simkin et al., 2015); its overexpression in tomato increased seed weight under suboptimal temperature conditions (Cai et al., 2022). The effect of expressing genes encoding bifunctional cyanobacterial enzymes, such as *SBPase* together with *FBPase*, has been explored in crops including tobacco, soybean, and lettuce (*Lactuca sativa*), resulting in greater RuBP regeneration efficiency. Additionally, co-expressing *Brassica napus SBPase* and cytosolic *FBPase* in tobacco increased the photosynthetic rate and resulted in substantial gains in biomass, plant height, stem diameter, and seed pod weight (Li et al., 2022). The redox regulation of *FBPase* and *SBPase* in regulating Calvin cycle efficiency is highlighted in section 3.3 of this review.

Transcriptional regulation also represents a powerful strategy for coordinating photosynthetic enhancement. Overexpressing the transcription factor gene *BRASSINAZOLE−RESISTANT 1* (*BZR1*) led to the upregulation of multiple downstream CBB genes including *Fructose−1,6−bisphosphate aldolase 1* (*FBA1*), *RCA1*, *Fructose−bisphosphatase 5* (*FBP5*), and *Phosphoglycerate kinase 1* (*PGK1*), resulting in improved photosynthetic performance and biomass accumulation in tomato (Yin et al., 2022). Such approaches leverage endogenous regulatory networks to drive balanced increases in pathway flux, mitigating stoichiometric and regulatory constraints often associated with constitutive expression of individual genes (**Table 3**). Advances in synthetic promoter design, gene stacking, and CRISPR-based genome editing have further enabled the fine-tuned expression of genes encoding key CBB enzymes such as *SBPase, PRK*, and *FBPA* in crops such as camelina (*Camelina sativa*), tobacco, and wheat (De Porcellinis et al., 2018; Simkin et al., 2019). These technologies facilitate precise spatial and temporal gene expression, enhancing pathway coordination while minimizing unintended metabolic penalties.

**Table 3:** Enhancing RuBP regeneration to improve photosynthetic capacity.

Organism	Engineering strategy	Observed photosynthetic & yield outcomes	References
Arabidopsis	Overexpression of *SBPase* (alone or with *FBPase*)	Enhanced RuBP regeneration; increased photosynthetic rate and growth	(Simkin et al., 2017a)
Tobacco	Co-overexpression of *SBPase*, *FBPase*, and *PRK*	Higher RuBP regeneration; higher CO_2_ fixation and yield	(Simkin et al., 2015)
Cyanobacteria, tobacco	Expression of cyanobacterial *SBPase*, *FBPase*, alone or together	Increased photosynthesis and biomass; stable yield under stress conditions	(Miyagawa et al., 2001) (Ichikawa et al., 2010)
Arabidopsis	Overexpression of the phosphoribulokinase gene (*PRK*)	Enhanced RuBP regeneration and carbon turnover; higher photosynthesis and seed yield	(Elena López-Calcagno et al., 2017)
Tobacco, Arabidopsis	Overexpression or modulation of *CP12*	Altered PRK and GAPDH activities; higher stress tolerance	(Howard et al., 2011) (Elena López-Calcagno et al., 2017)
potato	Expression of the glycolate dehydrogenase gene (*GDH*)	Increased CO_2_ assimilation and biomass	(Nölke et al., 2014)
Rice	*ZmPEPC* overexpression	55% increase in the photosynthetic rate under light saturation and 50% increase in carboxylation efficiency	(Demao et al., 2001)

Because RuBP regeneration is tightly coupled to the supply of ATP and NADPH, several studies have focused on augmenting photosynthetic electron transport to relieve energetic constraints. Co-expressing *SBPase* or *FBPase* with the gene encoding Cytochrome C_6_, an efficient algal electron carrier that enhances electron flow from the cytochrome b_6_f complex to PSI, resulted in greater carbon assimilation, yield, and water-use efficiency in tobacco under field conditions (Lopez-Calcagno et al., 2020). Transglutaminase (TGase) positively regulates photosynthetic performance in tomato. Using both overexpression and CRISPR/Cas9-mediated knockout approaches, Zhong et al. (2019) demonstrated that TGase enhances CO_2_ assimilation, RuBP carboxylation, and RuBP regeneration by promoting the expression and activation of genes encoding key CBB cycle enzymes, including Rubisco, RCA, and FBPase, without altering total Rubisco abundance.

### 3.3 Optimization of light usage and redox balance

In addition to biochemical enzyme capacity, RuBP regeneration (J*_max_*) is strongly constrained by the efficiency of photochemical reactions and redox regulation in chloroplasts, which determine both the energy supply and activation state of Calvin cycle enzymes (Raines, 2003). In natural canopies, plant architecture and growth patterns are shaped by environmental factors such as light intensity and angle. Efficient and uniform absorption of photosynthetically active radiation (PAR) is essential for driving water splitting and sustaining electron transport, both of which underpin photosynthetic performance. Adjusting the size and composition of light-harvesting antenna complexes provides another means for balancing light absorption and utilization. Loss of function of SIGNAL RECOGNITION PARTICLE 43 (SRP43), a molecular chaperone required for integrating light-harvesting chlorophyll-binding proteins (LHCPs) into the thylakoid membrane, reduced antenna size, improved excitation balance between PSI and PSII, and enhanced photosynthetic efficiency in tobacco under field-like conditions (Kirst et al., 2018). However, excessively small antennas can compromise photoprotection under high or fluctuating light conditions. To mitigate this trade-off, synergistic photoprotection systems have been developed. Engineering faster NPQ kinetics was achieved in plants via the coordinated overexpression of three genes comprising the VPZ module: *Violaxanthin De-epoxidase* (*VDE*), *PS II Subunit S* (*PsbS*), and *Zeaxanthin Epoxidase* (*ZEP*); this module accelerates both the induction and relaxation of energy dissipation, improving light-use efficiency under fluctuating light (Kromdijk et al., 2016; Milburn et al., 2025). While this approach increased biomass in tobacco, responses in tomato and Arabidopsis have been more variable, highlighting the need for species-specific optimization (Garcia-Molina and Leister, 2020; Lehretz et al., 2022).

Maintaining the redox balance of chloroplasts is equally critical for sustaining RuBP regeneration, specifically under environments imposing oxidative stress. Cyclic electron flow around PSI contributes to photoprotection by generating additional ATP without further reducing the electron transport chain, thereby adjusting the ATP/NADPH ratio to match CBB cycle demands under fluctuating light conditions (Yamori et al., 2016; Chadee et al., 2021). Alternative electron sinks provide complementary safeguards. Recent studies report that genes encoding flavodiiron proteins (FLVs), which act as electron safety valves in cyanobacteria and algae, have been successfully expressed in land plants (Leister, 2023). Expressing FLV genes in Arabidopsis alleviated PSI over-reduction and photoinhibition under fluctuating light conditions by diverting excess electrons (Gerotto et al., 2016; Yamamoto et al., 2016; Wada et al., 2018). Similarly, plastid terminal oxidase (PTOX) and mitochondrial alternative oxidase (AOX) act as dynamic electron sinks, preventing over-reduction of the photosynthetic apparatus and helping maintain ATP production without excessive NADPH accumulation (Stepien and Johnson, 2009; McDonald et al., 2011).

The thioredoxin (TRX) system in chloroplasts coordinates photochemical RuBP regeneration in plants, particularly under photooxidative stress conditions. Thioredoxins are small proteins capable of reducing disulfide bonds in target proteins, rendering them active or inactive. TRXs are grouped into the f, m, x, y, z, h, and o classes; class f, m, x, y, and z localize to chloroplasts. By contrast, NTRC (NADPH-dependent thioredoxin reductase C), TrxL2, and ACHTs constitute non-canonical chloroplast redox regulatory systems (Nikkanen and Rintamäki, 2014). The canonical activating TRXs (f and m) reduce and activate key CBB cycle enzymes such as FBPase, SBPase, GAPDH, PRK, ATP synthase, and MDH. In parallel, the NTRC thioredoxin system senses stromal NADPH levels to regulate enzyme activation and energy metabolism. By contrast, TRX-x and TRX-y, together with TRX-like proteins (TrxL2 and ACHT), promote the oxidation of target proteins, resulting in their inactivation.

Functional studies have demonstrated that manipulating these TRX systems can significantly influence photosynthesis and plant growth (Nikkanen et al., 2017). Ectopic expression of *Arabidopsis NTRC* resulted in a substantial rise in the CO_2_ assimilation rate, higher PS-I efficiency under low light conditions, and lower NPQ under high-light conditions in transgenic *Arabidopsis* (Toivola et al., 2013). NTRC maintains the thiol–disulfide status of ATP synthase in the chloroplast, modulating the efficiency of ATP production (Carrillo et al., 2016). By coordinating the activation of antioxidant systems and CBB enzymes, NTRC prevents the over-reduction of the electron transport chain. This regulation of redox balance in the chloroplast stroma helps suppress reactive oxygen species formation under fluctuating light conditions (Kramer et al., 1990). The overall mechanism enhances light energy capture and its subsequent utilization for RuBP regeneration. On the other hand, overexpressing *TRX-f* in tobacco did not influence steady-state CO_2_ assimilation but significantly enhanced starch and soluble sugar accumulation, suggesting altered carbon partitioning (Sanz-Barrio et al., 2013). This hypothesis is strengthened by the finding that overexpressing *TRX-m* in tobacco reduced sugar and starch accumulation but enhanced amino acid and soluble protein accumulation, indicating that nitrogen metabolism was altered (Ancín et al., 2021).

TRX-like2 (TrxL2) and atypical Cys His-rich TRX (ACHT) are responsible for oxidizing their target proteins ATP synthase and FBPase (Yoshida et al., 2019; Yokochi et al., 2021). Consequently, overexpressing *ACHT2* in Arabidopsis led to oxidized chloroplast proteins, thereby inactivating CBB cycle enzymes in the light, thus inducing negative feedback on the photosynthetic electron transport chain (Fukushi et al., 2024). These context-dependent outcomes indicate that TRXs are not universal activators of photosynthesis, instead functioning in a complex variety of metabolite-redox interactions that alter electron transport, carbon assimilation, energy dissipation, and metabolic allocation. Future engineering efforts should move beyond simple overexpression to exploring the roles of TRX systems in the balanced activation and oxidation of photosynthetic proteins. Studies combining redox proteomics, metabolic flux analysis, and *in silico* system modeling may further clarify TRX-mediated carbon and nitrogen allocation, energy dissipation, and overall photosynthetic efficiency in crops.

## 4 Biochemical limitation: triose-phosphate utilization

During the CBB cycle, triose phosphates (TPs), primarily glyceraldehyde-3-phosphate (GAP) and dihydroxyacetone phosphate (DHAP), are produced and exported from the chloroplast via phosphate translocators in the inner chloroplast membrane. These TPs serve as precursors for starch biosynthesis within the chloroplast and for sucrose biosynthesis in the cytosol. The metabolic processing and utilization of these end products for plant growth, maintenance, and energy generation are collectively termed TPU. TPU represents a critical interface between carbon assimilation and downstream metabolic demands, linking carbon fixation in chloroplasts and source–sink balance throughout the plant (Sharkey, 1985; McClain and Sharkey, 2019).

Photosynthesis can become limited by TPU even when Vc_max_ and J_max_ operate near capacity. In A/Ci response curves (**Figure 1**), TPU limitation is typically observed at elevated C*i*, where further increases in C*i* fail to proportionally increase net assimilation; the curve instead reaches a plateau or even points downward (Sharkey, 1985; Laporte et al., 2001). This behavior reflects a biochemical bottleneck in the conversion of TPs into sucrose and starch relative to incoming carbon (McClain and Sharkey, 2019). When TP export and end-product biosynthesis are insufficient, inorganic phosphate (Pi) becomes sequestered in phosphorylated intermediates within the chloroplast, limiting ATP production and slowing photosynthesis (Pieters et al., 2001; Heldt and Piechulla, 2021). Because Pi recycling depends on efficient TP export and sucrose biosynthesis, TPU limitation is often described as Pi limitation (Paul and Foyer, 2001). This constraint is most pronounced under high sucrose-to-sink ratios, nutrient limitations, or restricted sink plasticity, underscoring TPU as a dynamic regulator of sink-source coordination (Ruiz-Vera et al., 2021). Temperature strongly modulates TPU limitation. When Rubisco-mediated carboxylation and RuBP regeneration approach saturation under elevated CO_2_, suboptimal temperatures can accentuate TPU constraints (Rogers et al., 2021). Early work showed that phosphate feeding stimulated photosynthesis in barley (*Hordeum vulgare*) plants at 10 °C but had little effect at 25 °C, indicating the temperature sensitivity of sucrose biosynthesis and TP utilization pathways (Leegood et al., 1988). Enzymes such as sucrose-phosphate synthase, nitrate reductase (as studied in tomato) (Jones et al., 1998), and cytosolic FBPase exhibit strong thermal sensitivity between ~4 and 12 °C, making TPU limitation more responsive to low and moderately low temperatures than when Vc_max_ and/or J_max_ limit photosynthesis (Sharkey and Bernacchi, 2011; Yang et al., 2016). Consistent with this idea, Arabidopsis showed TPU limitation at 15–25 °C but not at higher temperatures, whereas mutants impaired in TP export (*tpt-1*) exhibited TPU limitations across all temperatures tested, confirming the central role of TP export in determining TPU sensitivity (Yang et al., 2016).

Under optimal conditions (CO_2_ levels of 400–420 ppm), TPU limitation is often transient, and species with relatively low sink capacity (such as Arabidopsis) rarely show sustained TPU limitation because photosynthetic rates acclimate to match assimilate export and storage capacity (Yang et al., 2016; McClain, 2022). TPU effects are also uncommon at CO_2_ levels below 800 ppm and may be further minimized after long-term acclimation to elevated CO_2_ levels, restoring the balance between triose sugar production and its utilization, thereby alleviating TPU limitation (Leakey et al., 2009; Kumarathunge et al., 2019). Consequently, TPU has historically been viewed as a minor or conditional limitation and is frequently omitted or simplified in terrestrial biosphere models (Sharkey, 1985; Harley and Sharkey, 1991; Wullschleger, 1993; Busch et al., 2018). However, emerging evidence indicates that current terrestrial biosphere models of TPU often lack species-specific temperature responses and mechanistic links to source–sink regulation, limiting their predictive accuracy under changing climates (Ricciuto et al., 2018; McClain and Sharkey, 2019; Walker et al., 2021; McClain et al., 2023).

### 4.1 Efficient TPU requires active sinks and sucrose metabolism

TPU is closely integrated with whole-plant carbon partitioning and sugar signaling. Sustained TP export under favorable conditions maintains plastid Pi availability and supports high photosynthetic rates, particularly in plants with strong sink capacity and active sucrose catabolism (Busch and Sage, 2017; Fabre et al., 2020). Conversely, lower sink demand triggers feedback regulation. Perturbations in starch biosynthesis, such as altered ADP-glucose pyrophosphorylase (AGPase) activity, can repress sugar transport and carbon export (Müller-Röber et al., 1990; Chiou and Bush, 1998). Sugars also function as signaling molecules through trehalose 6-phosphate (T6P) and sucrose-non-fermenting 1-related protein kinase 1 (SnRK1)-mediated pathways, linking carbohydrate status to the transcriptional regulation of photosynthetic genes (Zhang et al., 2009; Fichtner et al., 2021). In Arabidopsis, lower expression levels of sucrose transporter genes induce retrograde signaling that downregulates the expression of nucleus-encoded photosynthetic components, including *RBCS*, *LHCP*s, and *RCA* (Moore et al., 1998; Fichtner et al., 2021).

Biotechnological strategies to alleviate TPU limitation have primarily focused on enhancing sink strength and improving TP metabolism and export (**Table 4**). Overexpressing the chloroplast-localized triose-phosphate isomerase (TPI) gene in rice increased the photosynthetic rate under elevated CO_2_ conditions (Suzuki et al., 2022). Manipulating transcriptional regulators such as *GLK* genes in maize enhanced carbohydrate accumulation and yield (Li et al., 2020). Engineering CBB cycle enzymes, including SBPase, FBPase, and PRK, improved RuBP regeneration and indirectly supported TPU in tobacco (Lefebvre et al., 2005; Simkin et al., 2019).

**Table 4:** TPU/Carbon export and sink-strengthening strategies for efficient Pi recycling and triose-phosphate usage.

Plant species	Engineering strategy	Observed photosynthetic & yield outcomes	References
Tobacco, Arabidopsis	Enhanced sucrose biosynthesis via cytosolic FBPase and SPS to increase TP usage and Pi recycling	Improved TPU, A_max_, and biomass	(Miyagawa et al., 2001; Uematsu et al., 2012)
Arabidopsis, potato	Upregulation of genes involved in starch biosynthesis (AGPase, starch synthases)	Increased sink capacity; sustained photosynthesis	(Stark et al., 1992; Bahaji et al., 2014)
Arabidopsis, tobacco	Overexpression of *TPT* (Triose-Phosphate/Phosphate Translocator gene)	Alleviated TPU limitation under high-CO_2_ and high-light conditions	(Schneider et al., 2002)
Arabidopsis	Overexpression of *TPT* and *PHT2;1*	Improved Pi import and photosynthetic efficiency	(Raju et al., 2024)
Tomato, Arabidopsis	Source–sink optimization (SWEETs, invertases)	Improved TP utilization and photosynthetic efficiency	(Paul and Foyer, 2001)
Tobacco, Arabidopsis	Multi-gene engineering (*SBPase*, *FBPase*, *TPT* overexpression)	Increased RuBP regeneration and TP export; higher assimilation and yield	(Simkin et al., 2017a)
Arabidopsis, rice, maize	Overexpression of *SWEET*	Enhanced sucrose transport to sink tissue; higher TPU and carbohydrate accumulation	(Chen et al., 2012; Sosso et al., 2015)
Tobacco	Overexpression of *AGPase*	Increased starch biosynthesis and TPU capacity	(Sonnewald and Fernie, 2018)
Tobacco	Upregulation of the sucrose synthase gene (*SUS*)	Enhanced sucrose cleavage: higher TPU and biomass	(Sonnewald and Fernie, 2018)

### 4.2 Connecting photosynthesis to metabolism: the importance of triose-phosphate export

Efficient photosynthesis requires tight coordination between carbon fixation in chloroplasts and metabolism in the cytosol. This integration is mediated by a network of phosphate metabolite transporters located in the inner chloroplast membranes, including Triose-phosphate/phosphate translocator (TPT), Xylulose 5-phosphate/phosphate translocator (XPT), Glucose 6-phosphate/phosphate translocator (GPT), and Phosphoenolpyruvate/phosphate translocator (PPT) in Arabidopsis (Flugge, 1998; Weber, 2004). These transporters regulate photosynthetic end-product partitioning, inorganic phosphate (Pi) recycling, and source–sink balance (**Figure 2**).

TPT is the central hub linking photosynthesis with cytosolic metabolism (Fliege et al., 1978). TPT mediates the exchange of stromal triose phosphates, glyceraldehyde-3-phosphate (GAP), and dihydroxyacetone phosphate (DHAP) or 3-phosphoglycerate (3-PGA) in exchange for cytosolic Pi (Flügge et al., 2003; Lee et al., 2017). This antiporter mechanism is essential for maintaining chloroplastic Pi availability, sustaining ATP biosynthesis, and supporting TPU. By linking carbon export from the chloroplast with cytosolic sucrose biosynthesis, TPT prevents the feedback inhibition of photosynthesis by facilitating continuous triose-phosphate export and Pi availability in stroma. Functional studies have underscored the importance of TPT in balancing carbon flux between the chloroplast and cytosol. Overexpressing Arabidopsis *TPT* (*AtTPT*), particularly in combination with the gene encoding cytosolic FBPase, resulted in enhanced CO_2_ assimilation, sucrose accumulation, biomass production, and Pi recycling (Cho et al., 2012; Lee et al., 2014). *AtTPT* overexpression also rescued the metabolic and growth defects observed in a mutant lacking two plastidial GAPCs (*gapcp1 gapcp2*), restoring normal carbon flux (Flores-Tornero et al., 2017). Conversely, tobacco lines silenced for *TPT* via antisense expression showed lower sucrose levels and a diminished capacity to increase photosynthetic rates under elevated CO_2_ conditions, suggesting impaired carbon export (Häusler et al., 2000).

Notably, Arabidopsis *tpt* single mutants often display only mild growth phenotypes due to metabolic compensation via altered pathways and transporters (Walters et al., 2004). However, double mutants lacking both TPT and XPT exhibit severe growth retardation and impaired photosynthesis, highlighting functional redundancy between these two plastidial phosphate translocators (Hilgers et al., 2018). Recent work further suggested that TPT influences sugar-responsive gene expression and carbon signaling, even when photosynthetic gene expression appears unchanged (Schmitz et al., 2014).

### 4.3 Transporters that maintain Pi homeostasis

Beyond TPT, additional plastidial transporters contribute to Pi homeostasis, metabolic flexibility, and carbohydrate partitioning, particularly under fluctuating environmental conditions, including Phosphate transporter-2 (PHT2), members of the PHT4 family, and Glucose-6-phosphate transporters (GPTs) (Versaw and Garcia, 2017). Arabidopsis *pht4* mutants exhibit reduced Pi levels, along with compromised ATP biosynthesis and reduced photosynthetic efficiency (Karlsson et al., 2015). Similarly, suppressing *OsPHT2;1* expression in rice reduced the Pi content in chloroplasts, limiting ATP biosynthesis activity and turnover of the CBB cycle (Liu et al., 2020). Conversely, heterologous expression of *GmPHT4* restored chloroplast Pi levels and enhanced photosynthetic capacity in the Arabidopsis *pht4;5* mutant (Liu et al., 2023).

GPT also plays important roles in coordinating plastidial carbon metabolism. In Arabidopsis, *GPT1* is ubiquitously expressed and is essential for the oxidative pentose phosphate pathway (OPPP) in non-green plastids (Kammerer et al., 1998; Niewiadomski et al., 2005; Baune et al., 2020). By contrast, *GPT2* expression is inducible and contributes to photosynthetic acclimation. GPT2 transports G6P and TPs; *GPT2* is upregulated under high-light conditions, facilitating the import of excess cytosolic carbon into chloroplasts for starch biosynthesis (Athanasiou et al., 2010; Dyson et al., 2015; Weise et al., 2019). The Arabidopsis *gpt2-1* mutant fails to increase photosynthetic capacity under high-light conditions, demonstrating the role of GPT2 in dynamic metabolic acclimation. Engineering GPT activity has also proven effective in storage tissues; overexpressing *GPT* and an adenylate translocator gene increased G6P availability, stimulated AGPase activity, and enhanced starch accumulation in potato (*Solanum tuberosum*) tubers (Jonik et al., 2012). XPT exchanges xylulose 5-phosphate and Pi across the chloroplast envelope and is thought to connect the cytosolic and plastidial pentose phosphate pathway (Eicks et al., 2002; Knappe et al., 2003). XPT provides metabolic flexibility and compensates for the loss of TPT, but its engineering potential remains unexplored.

### 4.4 Enhancing sucrose biosynthesis and export

Multiple biotechnological strategies that enhance sucrose transport, starch biosynthesis, and sink organ development improve carbon partitioning and yield under elevated light and CO_2_ conditions (Cho et al., 2012; Eom et al., 2015). For example, ectopic expression of the cytosolic fructose-1,6-bisphosphatase (cFBPase) gene increased cytosolic sucrose biosynthesis capacity in tobacco, facilitating the removal of excess TPs from chloroplasts and enhancing photosynthetic efficiency (Uematsu et al., 2012). Similarly, overexpressing sucrose transporter genes such as *AtSUC2* in Arabidopsis promoted efficient sucrose translocation from source leaves to sink tissues, relieving TPU limitations under elevated CO_2_ conditions (Srivastava et al., 2009; Wang et al., 2015).

Synthetic and transgenic approaches that directly enhance sink metabolism can reinforce source–sink coordination. Expressing a Pi feedback-insensitive form of *AGPase*, the *Shrunken 2* (*Sh2*) variant *Sh2r6hs*, significantly increased starch biosynthesis and sink strength in wheat, resulting in a 38% increase in seed weight per plant and more than 30% increase in total biomass compared to the wild type (Smidansky et al., 2002). Evidence from crop species strongly supports the role of sink strength in maintaining photosynthetic capacity under elevated CO_2_ conditions (Ruiz-Vera et al., 2017). Soybean genotypes with higher seed-setting capacity sustain greater photosynthetic activity and achieve larger yield gains under elevated CO_2_ conditions (Borrás et al., 2004). Cassava (*Manihot esculenta*), which possesses large tubers that act as powerful sinks, maintains high photosynthetic rates when grown under elevated CO_2_ (>600 ppm) conditions, while sugars and starch accumulate without changes in *V*_cmax_ or *J*_max_ (Ruiz-Vera et al., 2021). Likewise, rice cultivars with enhanced tillering capacity and higher grain numbers exhibited improved photosynthetic performance when CO_2_ was enriched to 200 µmol mol^−1^ above the ambient CO_2_ level (Hasegawa et al., 2013).

### 4.5 Sink removal downregulates photosynthesis

Conversely, experimentally reducing sink size leads to carbohydrate accumulation in source leaves and the suppression of photosynthesis. Panicle pruning causes a buildup of starch and sugars in rice leaves, triggering TPU limitation and photosynthetic downregulation (Fabre et al., 2019). Consistent with this observation, free-air CO_2_ enrichment (FACE) studies in which the concentration of CO_2_ was increased around vegetation in open-air plots have shown that rice cultivars with high spikelet density exhibit greater photosynthetic stimulation and enhanced yields under elevated CO_2_ conditions, underscoring the importance of genotypic sink plasticity for optimizing carbon-use efficiency (Hasegawa et al., 2013; Lv et al., 2020).

Genetic manipulation of carbon transport pathways further illustrated the importance of sink strength. Overexpressing sucrose transporter genes, including *LeSUT1* (*SlSUT1*) and *LeSUT2* (*SlSUT1*); *AtSWEET10–15*; and *OsSWEET11* and *OsSWEET14* in tomato, Arabidopsis, and rice, respectively, enhanced carbon partitioning and mitigated photosynthetic acclimation under elevated CO_2_ conditions (Osorio et al., 2014). Similarly, potato lines overexpressing a sucrose synthase gene exhibited greater starch accumulation in tubers and maintained higher photosynthetic rates and biomass under CO_2_ enrichment compared to the wild type (Baroja-Fernandez et al., 2009).

A central regulator linking sucrose availability to sink strength is the T6P signaling pathway. T6P functions as a proxy for sucrose status and modulates the activity of SnRK1, a master regulator of energy homeostasis (Schluepmann et al., 2003; Zhang et al., 2009). Elevated sucrose levels increase T6P concentrations, which inhibits SnRK1 activity and promotes anabolic pathways such as starch, lipid, and protein biosynthesis, thereby strengthening sink demand (Nunes et al., 2013). The manipulation of T6P metabolism has therefore emerged as a promising strategy to enhance sink strength and photosynthetic performance. Overexpressing a bacterial T6P synthase gene (*otsA*) increased T6P contents in tobacco, resulting in darker green leaves and a higher photosynthetic rate, whereas T6P depletion via expression of the T6P phosphatase gene *otsB* led to paler leaves with lower carbon assimilation (Pellny et al., 2002). The phloem-specific overexpression of a T6P synthase gene in maize reproductive tissues enhanced sucrose transport to developing kernels under drought stress by prolonging photosynthetic activity in source leaves (Oszvald et al., 2018). Similarly, genetic and chemical manipulation of T6P metabolism in both wheat and Arabidopsis increased grain/seed size and yield potential by reinforcing sink strength during reproductive development (Griffiths et al., 2016).

Beyond its role in carbon allocation, T6P signaling contributes to Pi homeostasis, which is critical for sustaining high photosynthetic flux. Indeed, lower T6P levels are associated with the accumulation of phosphorylated intermediates and impaired Pi recycling, potentially exacerbating TPU limitation. Enhancing T6P signaling may therefore facilitate Pi turnover and TP export, alleviating feedback inhibition and sustaining photosynthesis performance under elevated CO_2_ conditions (Paul and Pellny, 2003; Zhang et al., 2009).

## 5 Emerging tools and enabling technologies

Traditional approaches, such as selective breeding, domestication, optimization of canopy architecture, and agronomic management practices have produced measurable enhancements in photosynthetic performance. However, these improvements are often context dependent and influenced by specific environmental or management conditions (Ort et al., 2015; Kromdijk et al., 2016). Given the scale and urgency of challenges to global food security, there is a pressing need for more precise, targeted, efficient strategies to enhance photosynthesis. Recent advances in biotechnological tools have opened new avenues for understanding and manipulating photosynthesis at the molecular level, offering transformative potential for crop improvement (Wang et al., 2020). Emerging strategies include gene editing [e.g., clustered regularly interspaced short palindromic repeats (CRISPR)/CRISPR-associated nuclease 9 (Cas9) systems], multi-gene stacking, synthetic pathway engineering, chloroplast transformation, and systems-level modeling (Orr et al., 2017; Stewart et al., 2018; Nazari et al., 2024; Bukhat et al., 2025; Chen et al., 2025).

### 5.1 Breeding and transgene-free approaches

Leveraging natural genetic diversity in photosynthetic traits such as Rubisco kinetics, light-harvesting efficiency, and stomatal conductance provides a non-transgenic avenue for improving photosynthetic efficiency (Long et al., 2015; Faralli et al., 2019; Silva-Pérez et al., 2020). Because quantitative traits governing photosynthesis are genetically complex and usually controlled by multiple genes, genome-wide association studies (GWAS) and quantitative trait locus (QTL) mapping represent complementary approaches to identify the underlying loci (Ali et al., 2025). For example, in a study on rapeseed (*Brassica napus* subsp. *napus*) (Li et al., 2024), GWAS coupled with QTL mapping identified 21 stable QTL clusters associated with photosynthetic traits (net photosynthetic rate, stomatal conductance, Ci, and transpiration rate) and leaf morphology traits (petiole length, petiole angle, leaf length, leaf area, and leaf width) linked to enhanced photosynthesis. By introgressing these clusters using marker-assisted breeding and speed-breeding, breeders could design transgene-free ideotypes with enhanced photosynthetic efficiency. Another advanced transgene-free approach for enhancing photosynthetic efficiency is CRISPR-Cas9-based promoter editing. A recent example is the multiplexed CRISPR-Cas9 mutagenesis of noncoding sequences upstream of the rice (*Oryza sativa*) *PSBS1* gene. This approach increased PSBS1 protein abundance 2–3 fold and enhanced non-photochemical quenching (NPQ) capacity and water-use efficiency to the same extent as heterologous gene expression (Patel-Tupper et al., 2024). This scalable, transgene-free strategy could improve photosynthetic efficiency while facilitating commercialization, regulatory approval, and the incorporation of beneficial alleles into conventional breeding programs.

### 5.2 Systems biology and predictive modeling

Mathematical modeling has emerged as a powerful systems-biology tool for predicting and optimizing photosynthetic performance in crops (Sukhova et al., 2021). As photosynthesis operates via a complex metabolic network, intuitively designed engineering interventions often fail. The e-Photosynthesis model employs ordinary differential equations to mechanistically integrate complex photochemical reactions, carbon metabolism, and regulatory processes to simulate *in vivo* leaf photosynthesis (Zhu et al., 2007, 2013). This framework enables *in silico* optimization of key metabolic enzymes including the those involved in the CBB cycle, photorespiration, starch and sucrose biosynthesis, and triose-phosphate utilization (Zhu et al., 2013), allowing multiple hypothetical targets to be evaluated prior to experimental validation.

For example, an e-Photosynthesis model predicted 23 photosynthetic enzymes that might increase CO_2_ assimilation by 67% in potato (*Solanum tuberosum*). Although the simultaneous manipulation of 23 targets is impractical, overexpressing the genes encoding only three of these enzymes (Rubisco, FBP aldolase, and SBPase-sedoheptulose-1,7-bisphosphatase) led to a substantial (28%) increase in the photosynthetic rate (Vijayakumar et al., 2024). Deploying this model in tobacco (*Nicotiana tabacum*) and soybean (*Glycine max*) has already boosted photosynthesis under present and future predicted climate conditions (Rosenthal et al., 2011; Köhler et al., 2017; Lopez-Calcagno et al., 2020). e-Photosynthesis models were recently upgraded by incorporating crop-specific proteins and their kinetics, making it possible to predict optimal combinations of engineering targets tailored to specific climatic conditions. For instance, Vijayakumar et al. (2025) identified Rubisco and SBPase as key targets for improving CO_2_ assimilation in rice under low CO_2_ conditions as well as aldolase and PRK for present CO_2_ levels. However, for elevated CO_2_ levels expected in the future, the model predicted Rubisco, SBPase, FBPase, and transketolase as prime targets. Going forward, this modeling approach could be applied to diverse crop species and growth environments to identify biotechnological targets for modulated expression to help overcome key biochemical limitations of photosynthesis.

## 6 Concluding remarks and outlook

Improving the efficiency of C_3_ photosynthesis remains one of the most compelling scientific and agricultural challenges of the 21st century. Increasing photosynthetic carbon assimilation has direct implications for crop productivity, resource-use efficiency, nutritional security, and climate resilience in the face of rapidly changing environmental conditions. Although Rubisco has historically received the greatest attention as the principal bottleneck in C_3_ photosynthesis, the evidence described in this review clearly demonstrates that photosynthetic efficiency is governed by a highly integrated, dynamic network involving Rubisco-mediated catalysis, RuBP regeneration, electron transport, photorespiration, triose-phosphate utilization (TPU), source–sink coordination, redox regulation, and whole-plant metabolic balance. Consequently, future gains in photosynthetic performance will likely depend not on the optimization of a single component, but rather on coordinated multi-target engineering strategies that simultaneously address interconnected biochemical and physiological limitations.

Recent advances in synthetic biology, systems biology, genome editing, computational modeling, and high-throughput phenotyping have created unprecedented opportunities to redesign photosynthetic metabolism. However, despite substantial progress, several fundamental research gaps remain unresolved. Under Rubisco-limited conditions, improving carboxylation efficiency without compromising CO_2_/O_2_ specificity continues to pose a major biochemical constraint. Although naturally occurring Rubisco variants with improved kinetic properties have been identified, the structural and evolutionary trade-offs governing catalytic efficiency, specificity, and stability are still poorly understood. Furthermore, the molecular mechanisms regulating Rubisco assembly, turnover, carbamylation, and interactions with RCA under fluctuating temperature and light conditions remain incompletely characterized. A deeper understanding of chloroplast chaperone systems, post-translational modifications, and isoform-specific RCA thermotolerance will be essential for developing robust Rubisco engineering strategies that function reliably under field conditions.

Likewise, significant uncertainties remain regarding the regulation and coordination of RuBP regeneration. Increasing the abundance of CBB cycle enzymes alone does not necessarily improve carbon fixation unless electron transport capacity, ATP/NADPH supply, stromal redox homeostasis, and enzyme activation states are simultaneously optimized. Emerging evidence suggests that TRX- and NTRC-mediated redox regulation plays a central role in synchronizing photosynthetic metabolism with rapidly changing environmental conditions. However, the dynamic interactions among redox signaling, cyclic electron flow, photoprotection, and carbon metabolism are not yet fully resolved. Therefore, future research should focus on the integrated optimization of electron transport, ATP generation, photoprotective mechanisms, and redox regulation in parallel with CBB cycle engineering.

An equally important but often underappreciated challenge involves TPU limitation and sink capacity. Enhancing upstream photosynthetic processes without sufficient phosphate recycling, sucrose/starch biosynthesis, phloem loading, or sink development may ultimately create new metabolic bottlenecks that constrain carbon assimilation. This highlights a critical need to better understand how photosynthetic carbon metabolism is coordinated with nutrient availability, source–sink communication, developmental programming, and stress-signaling pathways. In particular, the integration of photosynthetic engineering with improvements in nitrogen-use efficiency, phosphorus utilization, water-use efficiency, and root architecture will likely determine the extent to which enhanced photosynthesis can be translated into sustainable yield gains under agricultural conditions.

Several major open questions also remain unresolved. First, the long-term metabolic and physiological consequences of simultaneously stacking multiple photosynthetic interventions remain largely unknown. Combining strategies such as Rubisco engineering, CCMs, synthetic photorespiratory bypasses, enhanced electron transport, and altered source–sink relationships may generate new bottlenecks, metabolic imbalances, or unintended trade-offs that are difficult to predict from single-gene studies. Second, most current studies are conducted under controlled laboratory or greenhouse environments, which poorly represent the fluctuating temperature, light, water, and nutrient conditions encountered in the field. Understanding how engineered photosynthetic pathways perform under dynamic environmental conditions, heat stress, drought, elevated CO_2_, and biotic stress remains a major research priority.

Another important frontier lies in improving the predictive capacity of systems-level photosynthetic models. Current crop simulation frameworks often lack sufficient integration of molecular regulation, metabolic flux, chloroplast signaling, developmental plasticity, and environmental interactions. The incorporation of multi-omics datasets, artificial intelligence-driven modeling, machine learning approaches, and digital twin crop platforms could greatly enhance the ability to predict emergent properties of engineered photosynthetic systems across diverse genetic backgrounds and agroecological environments. Such predictive frameworks will be invaluable for prioritizing engineering targets, minimizing unintended metabolic disruptions, and accelerating translational crop improvement.

Future progress will also require a broader shift from reductionist approaches toward holistic, multiscale engineering strategies that integrate chloroplast metabolism with whole-plant physiology, canopy architecture, rhizosphere interactions, and ecosystem-level performance. Importantly, improving photosynthetic efficiency alone may not guarantee increased crop productivity unless accompanied by enhanced sink strength, optimized nutrient acquisition, stress resilience, and favorable agronomic traits. Therefore, future research should focus on integrating photosynthetic engineering with breeding, phenomics, precision agriculture, and climate-resilient crop management practices.

Ultimately, overcoming the biochemical limitations of C_3_ photosynthesis will require interdisciplinary collaboration spanning molecular biology, plant physiology, structural biology, computational biology, agronomy, and environmental sciences. Although significant challenges remain, the convergence of advanced genome engineering technologies, synthetic metabolic design, high-resolution phenotyping, and systems-level modeling provides an unprecedented opportunity to bridge the long-standing gap between theoretical and realized photosynthetic efficiency. The coming decade may therefore represent a transformative period in which integrated photosynthetic engineering evolves from proof-of-concept studies into practical strategies for developing high-yielding, resource-efficient, climate-resilient crops capable of supporting global food and bioenergy demands in a changing world.
